# BiGKbhb: a bi-directional gated recurrent unit model for predicting lysine β-hydroxybutyrylation sites

**DOI:** 10.1186/s12864-025-12166-9

**Published:** 2026-01-21

**Authors:** Heba M. Elreify, Fathi E. Abd El-Samie, Moawad I. Dessouky, Hanaa Torkey, Said E. El-Khamy, Wafaa A. Shalaby

**Affiliations:** 1 Department of Electronics and Electrical Communication Engineering, Faculty of Electronic Engineering, Menoufia University, Menouf, 32952 Egypt; 2 Department of Information Technology, College of Computer and Information Sciences, Princess Nourah bint Abdulrahman University, P.O. Box 84428, Riyadh, 11671 Saudi Arabia; 3 Department of Computer Science and Engineering, Faculty of Electronic Engineering, Menoufia University, Menouf, 32952 Egypt; 4 Department of Computer Science, College of Computer Engineering and Sciences, Prince Sattam Bin Abdulaziz University, Al-Kharj, 16273 Saudi Arabia; 5 Department of Electrical Engineering, Faculty of Engineering, Alexandria University, Alexandria, Egypt

**Keywords:** Post-translational modifications, Β-hydroxybutyrylation, Kbhb, BLOSUM, Bidirectional gated recurrent unit, Protein language models

## Abstract

Post-Translational Modifications (PTMs) are covalent chemical alterations that occur after protein synthesis, critically regulating protein function, localization, and interactions. β-hydroxybutyrylation (Kbhb), a metabolically derived histone modification discovered in 2016, influences gene activation and cellular metabolism. While accurate PTM site identification is essential for understanding protein regulation and disease mechanisms, experimental approaches face significant limitations, including low modification abundance, high cost, and limited proteome coverage. Kbhb remains computationally underexplored, with only three existing prediction tools exhibiting modest accuracy and limited cross-species applicability. To address this gap, we developed BiGKbhb, a deep learning framework that depends on Bidirectional Gated Recurrent Units (BiGRU). With BiGKbhb, we systematically evaluate seven protein sequence encoding strategies, and compare six deep learning architectures using datasets from human, mouse, and fungal species. Results demonstrated that BLOSUM62 evolutionary encoding combined with BiGRU architecture achieves optimal performance, with BiGKbhb consistently achieving higher accuracy than those of existing methods with test set accuracies of 0.824, 0.832, and 0.871 for human, mouse, and fungal balanced datasets, respectively, with corresponding Area Under Curve (AUC) values of 0.920, 0.902, and 0.945, while additional evaluation on imbalanced datasets confirmed model robustness under realistic conditions. Cross-species analysis revealed enhanced transferability of the general multi-species model, and statistical validation confirmed significant improvements over existing predictors (*p* < 0.05). These findings contribute a robust computational tool for Kbhb prediction and provide insights into sequence determinants of this important modification across evolutionarily diverse species.

**Supplementary Information:**

The online version contains supplementary material available at 10.1186/s12864-025-12166-9.

## Introduction

The PTMs represent one of the most fundamental and sophisticated mechanisms by which cells regulate protein function, localization, stability, and interactions. These covalent chemical modifications occur after protein synthesis and dramatically expand the functional diversity of the proteome beyond what is encoded in the genome [1]. The PTMs serve as dynamic regulatory switches that allow cells to rapidly respond to environmental changes, developmental cues, and pathological conditions without requiring new gene expression [2].

The importance of PTMs in cellular biology cannot be overstated, as they control virtually every aspect of protein biology, including enzyme activity, protein-protein interactions, subcellular localization, protein degradation, and signal transduction pathways [3]. Dysregulation of PTM processes has been implicated in numerous human diseases, including cancer, neurodegenerative disorders, metabolic diseases, and aging-related pathologies, making PTM research crucial for understanding disease mechanisms and developing therapeutic interventions [4].

The PTMs occur through the coordinated action of specific enzymes that catalyze the addition or removal of chemical groups to target amino acid residues. These modifications can happen co-translationally during protein synthesis or post-translationally after protein folding and assembly [5]. The timing of PTM events is precisely regulated, and it often occurs in response to specific cellular conditions, such as changes in metabolite concentrations, stress conditions, cell cycle progression, or signal transduction cascades. Many PTMs are reversible, allowing for dynamic regulation of protein function in response to changing cellular needs. The enzymes responsible for PTMs, including kinases, phosphatases, acetyltransferases, deacetylases, methyltransferases, and demethylases, are themselves subject to regulation, creating complex regulatory networks that fine-tune cellular responses [6].

The landscape of PTMs is remarkably diverse, with over 400 different types of modifications identified to date [7]. Major categories include phosphorylation, the most extensively studied PTM that regulates protein activity and signalling pathways; acetylation, which plays crucial roles in gene regulation and metabolism; methylation, particularly important in epigenetic regulation and chromatin remodelling; ubiquitination that is essential for protein degradation and cellular localization; SUMOylation (Small Ubiquitin-like Modifier) that is involved in transcriptional regulation and nuclear transport; glycosylation that is critical for protein folding and cell-cell recognition [8]; hydroxylation that is important in collagen formation and hypoxia response; and nitrosylation which modulates protein function under oxidative stress conditions. Each modification type exhibits distinct chemical properties, regulatory mechanisms, and biological functions, contributing to the complexity of cellular regulation [9, 10].

Among the twenty standard amino acids, lysine residues serve as particularly important targets for PTMs due to their positively-charged side chain and chemical reactivity. Lysine modifications play central roles in diverse biological processes, with lysine acetylation and methylation being extensively characterized by their roles in chromatin regulation and gene expression [11]. Other significant lysine modifications include ubiquitination, which targets proteins for degradation or alters their localization; crotonylation, which is associated with active transcription; succinylation, which is linked to metabolic regulation; malonylation, which is important in fatty acid metabolism; glutarylation, which is involved in lysine catabolism; and the more recently discovered Kbhb, which represents a novel histone mark associated with gene activation and metabolic regulation. Kbhb involves the addition of a β-hydroxybutyryl group to lysine residues, neutralizing their positive charge, altering electrostatic interactions, and potentially creating binding sites for reader proteins. Initially characterized in histones, Kbhb has also been detected in non-histone proteins, showing broader regulatory roles in cellular processes [12].

Importantly, Kbhb must be distinguished from the structurally-related but chemically-distinct 2-hydroxyisobutyrylation (Khib). Although both are metabolism-derived lysine acylations, they differ in chemical structure, enzymatic machinery, and biological function. Khib, marked by the addition of a 2-hydroxyisobutyryl group (+ 86 Da), is widely distributed across histone and non-histone proteins in diverse organisms, where it regulates chromatin dynamics, transcription, and metabolism, and is implicated in diseases such as cancer and neurodegenerative disorders [13]. Since our most recent work focused on developing computational tools for Khib site prediction [14], we emphasize this distinction here to clarify the scope of this presented study.

Experimental identification of PTM sites traditionally relies on mass spectrometry-based proteomics approaches, including tandem Mass Spectrometry (MS/MS), which provides high-resolution identification and quantification of modified peptides [15]. However, these methods face significant challenges: low abundance of many modifications, dynamic nature of PTMs, requirement for specialized enrichment techniques, high cost and time requirements, technical complexity requiring specialized expertise, and difficulty in achieving comprehensive proteome-wide coverage. Sample preparation processes can introduce artifacts or lead to loss of chemically-unstable modifications, and modifications occurring under specific conditions or in low-abundance proteins may be missed entirely [16].

These experimental limitations have driven the development of computational frameworks for PTM prediction. Such frameworks offer distinct advantages: they enable genome-wide screening of potential modification sites, reducing the search space for experimental validation; provide cost-effective and scalable alternatives to labour-intensive techniques; support rapid hypothesis generation and cross-species comparative analysis; and facilitate exploration of conditions that are experimentally challenging to replicate [17]. Computational predictions also recommend the design of site-directed mutagenesis, prioritize candidates for functional studies, and advance our understanding of sequence motifs that dictate modification specificity.

Recent years have witnessed substantial advances in the computational prediction of lysine PTMs, driven by machine learning and deep learning methodologies. A survey by Qin et al. (2024) [12] identified 166 tools targeting 11 lysine modification types. These include notable predictors for acetylation (e.g., LAceP [18] and DeepDA-Ace [19]), methylation (e.g., Methyl-GP [20], MuLan-Methyl [21] and MethEvo [22]), ubiquitination (e.g., UbiSite [23], DeepUbi [24]), crotonylation (e.g., iCrotK-PseAAC [25], Adapt-Kcr [26] and BERT-Kcr [27]), succinylation (e.g., Deep_KsuccSite [28], HybridSucc [29] and LMsuccsite [30]), glutarylation (e.g., BiPepGlut [31] and [32]), and malonylation (e.g., SEMal [33] and Mal-light [34]).

However, certain modifications, such as Kbhb, remain computationally underexplored, indicating ongoing challenges and opportunities for innovation. Kbhb is a metabolically derived histone modification identified in 2016 [35], with emerging roles in gene activation, metabolism, and stress responses. Despite its biological relevance, Kbhb remains underrepresented in computational research, with only three prediction tools currently available: iBhb-Lys [36], which employs a combination of multiple feature encoding strategies with fuzzy support vector machines; KbhbXG [37], which utilizes the XGBoost gradient boosting framework; and SLAM [38], which represents the first deep-learning-based method for Kbhb site detection, incorporating structural information for structure-guided identification and achieving promising performance.

Despite these initial efforts, significant methodological gaps persist: (1) limited systematic evaluation of modern protein language models for Kbhb prediction, (2) absence of comprehensive cross-species transferability analysis and (3) lack of standardized architectural comparisons using identical datasets.

This study addresses the critical gap in Kbhb prediction through several key contributions that advance the field of computational proteomics:

- We conducted a comprehensive evaluation of seven protein sequence encoding strategies spanning four distinct categories: embedding-based approaches (Evolutionary Scale Modeling (ESM) [39], ProtBERT [40], and ProtGPT2 [41]), sequence context-based encoding (one-hot), physicochemical properties-based methods (Composition, Transition, Distribution (CTD) descriptors [42] and Amino Acid Properties (AAP)) [43], and evolutionary representation (BLOcks SUbstitution Matrix 62 (BLOSUM62)) [44].
- We evaluated six deep learning architectures: (Deep Neural Network (DNN) [45], One-Dimensional Convolutional Neural Network (1DCNN) [46], Long Short-Term Memory (LSTM) [47], Bidirectional Long Short-Term Memory (BiLSTM) [48], Gated Recurrent Unit (GRU) [49] and BiGRU [50], establishing BiGRU as the optimal architecture for Kbhb site prediction.
- We proposed a novel BiGRU-based architecture (BiGKbhb) that integrates BLOSUM62 encoding with deep learning components such as global max-pooling and dropout, reflecting a biologically-informed and effective design for PTM site prediction.
- We benchmarked BiGKbhb against existing Kbhb predictors, iBhb-Lys and KbhbXG, demonstrating consistent and statistically-significant performance improvements across all datasets, validated through DeLong’s test [51] with Bonferroni correction [52].
- We conducted cross-species generalization assessment across human, mouse, and fungal datasets. Additionally, we used *t*-distributed Stochastic Neighbor Embedding (*t*-SNE) [53] analysis for feature learning visualization.

The remainder of this paper is organized as follows. The next section outlines the methodology. After that, comprehensive results are presented. The last section gives the conclusion of the study by summarizing key contributions and their implications for computational proteomics and PTM prediction, while addressing current limitations and proposing directions for future research.

## Methods

**Fig. 1:**
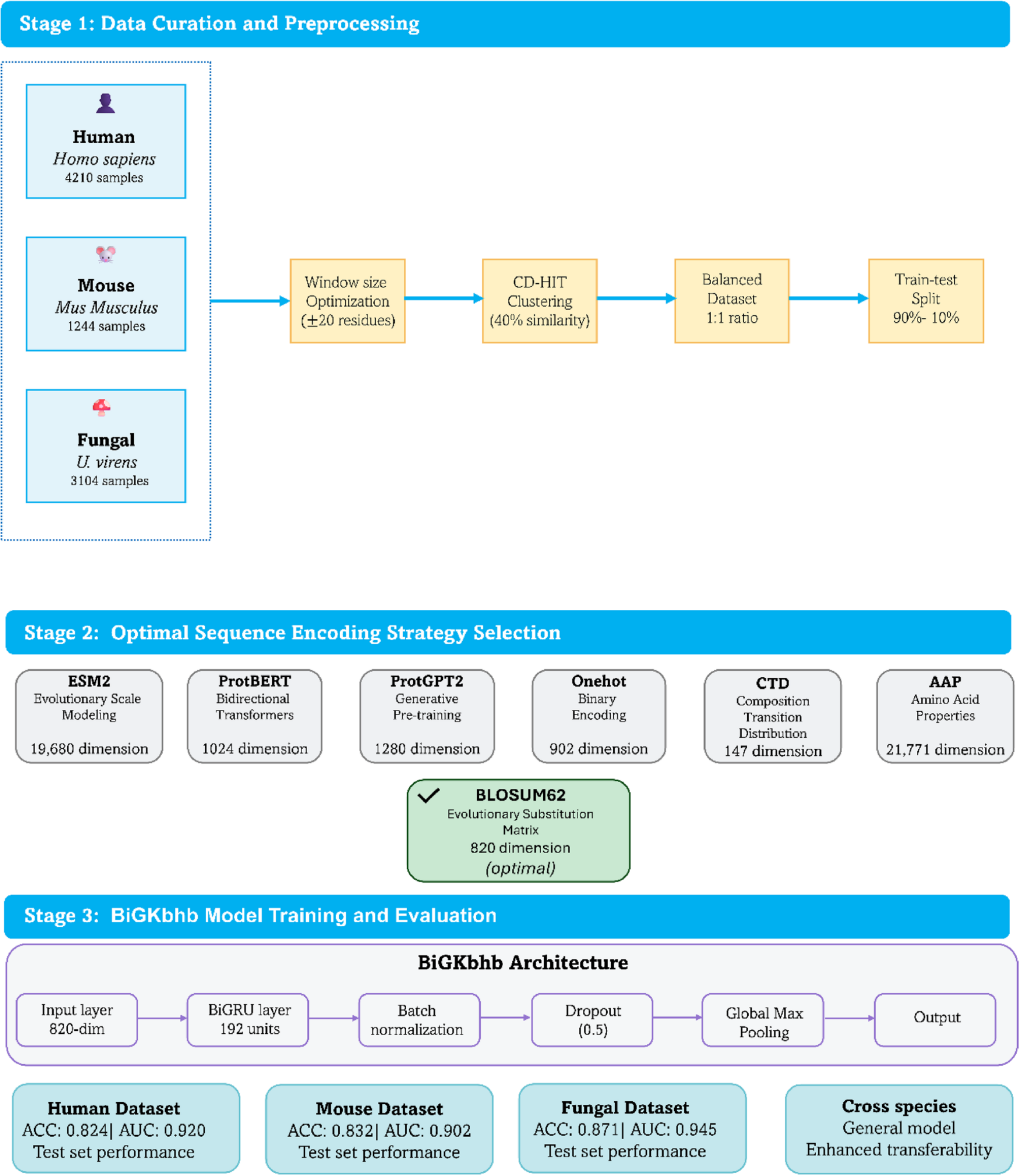
Workflow of the BiGKbhb framework showing the three-stage methodology: (i) Data curation from three evolutionarily diverse species with preprocessing steps including window size optimization, clustering-based balancing, and dataset partitioning; (ii) Systematic evaluation of seven protein sequence encoding strategies with BLOSUM62 identified as optimal; (iii) BiGKbhb model architecture implementation with bidirectional GRU processing and performance evaluation across all datasets demonstrating superior predictive capabilities

A systematic three-stage workflow is illustrated in Fig. 1, consisting of data curation from three species, optimal sequence encoding strategy selection, and classification using the proposed BiGRU-based model (BiGKbhb).

###  Dataset

Experimentally-validated Kbhb modification sites were compiled from the published literature for three evolutionary diverse species: Homo sapiens (human) [54], Mus musculus (mouse) [55], and Ustilaginoidea virens (fungal) [56]. The initial datasets comprised 3248, 840, and 2204 verified Kbhb sites for human, mouse, and fungal species, respectively, as detailed in Table 1.

**Table 1:** Distribution of samples in the datasets used for Kbhb site prediction

Dataset	Number of positive samples before clustering	Number of proteins	Number of positive samples after clustering	Total samples	Ref.
Human	3248	1397	2105	4210	[54]
Mouse	840	429	622	1244	[55]
*U. virens* (fungal)	2204	852	1552	3104	[56]
General (Merged species)	6292	2678	4279	8558	

Rather than analyzing complete protein sequences, we extracted peptide sequences centred on modified lysine residues using a custom Python [57] script that queried UniProt and UniParc [58] databases with protein accession numbers and lysine position data. Window size optimization was conducted systematically using the human dataset as a representative case. Nine different window sizes (15–51 amino acids, corresponding to ± 7 to ± 25 residues flanking the central lysine) were evaluated using 10-fold cross-validation AUC scores [59]. The 41-residue window (± 20 flanking residues) achieved optimal performance and was adopted for all subsequent analyses. Peptides from lysine residues near protein termini were padded with “X” symbols to maintain uniform sequence length.

Non-modified lysine residues serving as negative samples were extracted from the same proteins containing verified Kbhb sites, excluding positions with reported modifications. This strategy generated substantially larger negative sample pools (75,470, 16,739, and 23,039 for human, mouse, and fungal datasets, respectively) compared to positive samples.

To eliminate sequence redundancy and minimize homology that could lead to model overfitting, we implemented a clustering-based approach using Cluster Database at High Identity with Tolerance (CD-HIT) [60] with a 40% sequence similarity threshold [61–63]. This procedure grouped similar sequences into clusters, retaining one representative sequence per cluster to eliminate redundancy. The clustering reduced positive samples to 2105, 622, and 1552 representative clusters for human, mouse, and fungal datasets, respectively. Negative samples were similarly clustered, yielding 16,900, 3796, and 6372 clusters. To address class imbalance while preserving the biological relevance established through redundancy removal, an equal number of negative clusters were randomly selected to match positive clusters, creating balanced datasets with 1:1 positive-to-negative ratios.

The final balanced datasets contained 4210, 1244, and 3104 samples for human, mouse, and fungal species, respectively. Each dataset was partitioned into training (90%) and independent test (10%) sets. Training sets underwent 10-fold cross-validation for model optimization, while test sets were reserved exclusively for final performance evaluation to ensure an unbiased assessment of model generalization capabilities.

To further evaluate model robustness under realistic conditions, we also assessed BiGKbhb performance on the original imbalanced datasets without artificial balancing (see Appendix A).

### Protein sequence encoding strategies

Four distinct categories encompassing seven specific encoding approaches were employed to evaluate the optimal encoding strategy for Kbhb site identification. The dimensional characteristics and computational requirements of each encoding method are summarized in Table 2, with encoding time calculations performed on a standard desktop system with an AMD Ryzen 5 7520U processor (2.80 GHz), 16.0 GB RAM, using the general dataset as the largest dataset to assess comprehensive computational efficiency.

**Table 2:** Protein sequence encoding strategies and their dimensional characteristics

Encoding method	Dimension	Encoding time (sec)
ESM2	19,680 (41 × 480)	842.38
ProtBERT	1024	5,072
ProtGPT2	1280	5,053
Onehot	902 (41 × 22)	0.18
CTD	147	0.21
AAP	21,771 (41 × 531)	35.53
BLOSUM62	820 (41 × 20)	0.65

#### Embedding-based encoding

##### ESM2 

represents a family of transformer-based protein language models that leverage large-scale unsupervised learning on evolutionary sequences to capture fundamental patterns of protein structure and function. These models employ a bidirectional transformer architecture trained on millions of protein sequences from diverse organisms, enabling them to learn rich representations of amino acid relationships and evolutionary constraints [64]. The ESM2 framework utilizes masked language modeling objectives, where random amino acids are masked during training, forcing the model to predict missing residues based on the surrounding sequence context. This approach allows the models to internalize complex dependencies between amino acids at varying distances within protein sequences, resulting in embeddings that capture both local structural motifs and long-range evolutionary relationships essential for understanding protein modification sites [56].

The ESM2 models generate contextualized per-residue embeddings by processing protein sequences through multiple transformer layers, where each amino acid position receives a dense vector representation that incorporates information from the entire sequence context. During inference, input protein sequences are tokenized and passed through the transformer architecture, with each layer refining the representations through self-attention mechanisms that weigh the importance of different sequence positions. The final hidden states from the last transformer layer serve as per-residue embeddings, capturing position-specific evolutionary and structural information. For tasks requiring fixed-length representations, ESM2 models generate sequence-level embeddings by applying pooling operations (typically mean pooling) across all per-residue embeddings, creating a single vector that summarizes the entire protein sequence, while preserving critical biochemical properties learned during pre-training.

The ESM2 model family encompasses six variants with increasing architectural complexity and representational capacity, where each model encodes specific architectural and training details [65]. The nomenclature follows the pattern “esm2_t[layers][parameters][dataset]”, where ‘t’ indicates the number of transformer layers, the numerical suffix represents the approximate parameter count, and ‘UR50D’ refers to the UniRef50 [66] training dataset with additional diversity filtering. As presented in Table 3, the available variants’ dimensions range from 320 to 5120, offering scalable embedding dimensions to accommodate for different computational constraints and application requirements. For this study, we employed esm2_t12_35M_UR50D, which generates 480-dimensional embeddings per residue, resulting in 41 × 480 feature vectors for our optimized window size of 41 amino acids. This variant was selected based on computational resource limitations, as larger models with higher-dimensional embeddings (640 + dimensions) exceeded our available CPU processing capacity. It still provides sufficient representational power for accurate Kbhb site prediction.

**Table 3:** ESM2 model variants and corresponding embedding dimensions

Model	Embedding dimension
esm2_t6_8m_UR50D	320
esm2_t12_35M_UR50D	480
esm2_t30_150m_UR50D	640
esm2_t33_650m_UR50D	1280
esm2_t36_3b_UR50D	2560
esm2_t48_15b_UR50D	5120

##### ProtBERT 

represents a domain-specific adaptation of the Bidirectional Encoder Representations from Transformers (BERT) architecture, specifically designed for protein sequence analysis through transfer learning from natural language processing methodologies. Unlike ESM models that were developed specifically for protein analysis, ProtBERT adapts the original BERT framework by treating amino acids as discrete tokens analogous to words in natural language, leveraging the proven success of BERT in capturing bidirectional contextual relationships [67]. The model was pre-trained on large-scale protein databases using the same masked language modeling objective as BERT, but with protein-specific tokenization and vocabulary that accounts for the unique properties of amino acid sequences and their evolutionary constraints.

ProtBERT generates fixed embeddings by applying pooling operations to the final hidden states across all sequence positions, producing a single consolidated vector representation that captures global sequence characteristics. Several ProtBERT variants are available with different architectural configurations, training datasets, and tokenization schemes, including ProtBERT-BFD trained on the Big Fantastic Database [68], and ProtBERT-UniRef trained on UniRef datasets [69], offering flexibility in model selection based on specific application requirements and computational constraints. For this study, we employed fixed embeddings generated by ProtBERT, which provide a unified 1024-dimensional representation of the entire 41-residue peptide sequence as a single feature vector offering computational efficiency, while maintaining the model capacity to encode protein-specific evolutionary and structural patterns essential for accurate Kbhb site identification.

##### ProtGPT2 

is an adaptation of the Generative Pre-trained Transformer 2 (GPT-2) autoregressive language model architecture, specifically fine-tuned for protein sequence generation and representation learning. In contrast to bidirectional models such as ESM and ProtBERT, which utilize masked language modeling, ProtGPT2 employs a unidirectional transformer decoder architecture that processes amino acid sequences in a left-to-right fashion. During pre-training, it adopts a causal language modeling objective, learning to predict the next amino acid conditioned on the preceding residues [67]. This enables the model to capture context-dependent sequential dependencies and amino acid transition patterns reflective of natural protein evolution.

Although ProtGPT2 is inherently designed for sequence generation, fixed-length protein embeddings can be derived by extracting representations from the final transformer layer and applying pooling operations across the sequence length, producing a consolidated vector that encapsulates the learned sequential patterns and compositional features of the input protein sequence. Several ProtGPT2 variants are available, including models trained on different protein databases with varying architectural parameters, such as ProtGPT2-small, ProtGPT2-medium, and ProtGPT2-large, each offering different trade-offs between model complexity and computational requirements. In our implementation, we utilized ProtGPT2 fixed embedding approach to generate 1280-dimensional feature vectors representing each peptide sequence, capitalizing on the model autoregressive learning to capture sequential amino acid dependencies and generative patterns that allow Kbhb modification site recognition.

#### Sequence context-based encoding

##### One-hot 

encoding is a fundamental sequence representation method that transforms protein sequences into binary matrices by creating a sparse vector representation for each amino acid position. This approach converts each amino acid into a binary vector where only one element is set to 1 (indicating the presence of that specific amino acid) while all other elements remain 0, creating a straightforward yet effective method for representing categorical amino acid information in a format suitable for machine learning algorithms. One-hot encoding preserves the positional information of amino acids within the sequence while maintaining complete independence between different amino acid types, making it particularly valuable for capturing local sequence patterns and positional preferences around modification sites.

In our implementation, each 41-residue peptide sequence is transformed into a 41 × 22 binary matrix, where each row corresponds to a specific position within the peptide and each column represents one of the 22 possible characters. The 22-dimensional vectors account for the 20 standard amino acids (A, C, D, E, F, G, H, I, K, L, M, N, P, Q, R, S, T, V, W, Y), the ambiguous amino acid selenocysteine (U), and the unknown amino acid placeholder (X) used for padding positions near protein termini. This encoding strategy generates a comprehensive 902-dimensional feature vector that explicitly captures both the amino acid identity and positional context essential for identifying sequence determinants of Kbhb modification sites.

#### Physicochemical-properties-based encoding

##### CTD 

encoding is a comprehensive feature extraction method designed to capture the physicochemical properties of protein sequences through three distinct yet complementary descriptors: Composition (C), Transition (T), and Distribution (D). This method classifies the 20 standard amino acids into predefined groups based on shared physicochemical characteristics such as hydrophobicity, polarity, charge, and molecular size, enabling the extraction of higher-order sequence patterns that reflect functional and structural constraints. Amino acids are typically grouped under various physicochemical schemes, including hydrophobic versus hydrophilic, charge-based (positive, negative, neutral), polarity (polar, non-polar), and size-based (small, medium, large) classifications. These groupings allow for a multidimensional characterization of protein sequences from multiple physicochemical perspectives.

The Composition descriptor quantifies the relative frequency of each amino acid group within the sequence, providing a global overview of its physicochemical makeup. The Transition descriptor measures the frequency of transitions between different amino acid groups along the sequence, thereby capturing local patterns and tendencies for certain properties to alternate or cluster. The Distribution descriptor characterizes the positional distribution of each amino acid group by calculating statistical landmarks such as the positions of the first, 25th percentile, 50th percentile (median), 75th percentile, and last occurrences, offering insights into the spatial organization of physicochemical features across the sequence.

In this study, CTD encoding was applied to 41-residue peptide sequences using seven physicochemical grouping schemes: (1) hydrophobicity, (2) normalized van der Waals volume, (3) polarity, (4) polarizability, (5) charge, (6) secondary structure propensity, and (7) solvent accessibility. Each scheme partitions the 20 standard amino acids into 3 groups, resulting in 7 × 3 = 21 distinct physicochemical groups. The Composition descriptor yields 21 features (one per group). The Transition descriptor contributes 21 features, representing the frequency of group-to-group transitions. The Distribution descriptor produces 105 features (5 positional metrics × 21 groups). Altogether, these components are concatenated into a 147-dimensional CTD feature vector (21 + 21 + 105 = 147), providing a robust quantitative representation of the physicochemical organization within each peptide.

##### AAP 

encoding is derived from the AAindex database, which represents a comprehensive collection of numerical indices characterizing the diverse physicochemical and biochemical properties of amino acids, originally developed at the Genome Information Center of Japan. Each AAindex entry consists of 20 numerical values corresponding to specific properties of the standard amino acids, derived from published experimental and theoretical studies encompassing hydrophobicity scales, secondary structure propensities, charge distributions, molecular volumes, and various other structural and chemical characteristics. The database has evolved significantly since its initial release, expanding from 437 indices to the current collection of 566 entries across multiple releases, providing a rich repository of amino acid property data essential for computational biology applications.

In this study, we downloaded the complete AAindex1 database and implemented a systematic filtering process to ensure data quality and completeness. Of the 566 indices reported in the database, our parsing algorithm successfully extracted 531 properties that contained complete numerical values for all 20 standard amino acids, with 35 entries excluded due to missing or incomplete data that could compromise the encoding integrity. Since the AAindex database only provides values for the 20 standard amino acids, non-standard residues and padding characters required special handling: both padding characters (X) introduced near protein termini and selenocysteine (U) were assigned zero values across all 531 properties to explicitly indicate the absence of characterized physicochemical data for these positions. Each 41-residue peptide sequence was transformed into a comprehensive feature matrix of dimensions 41 × 531, subsequently flattened to generate 21,771-dimensional feature vectors that capture the complete spectrum of position-specific physicochemical characteristics surrounding each potential Kbhb modification site.

#### Evolutionary encoding

BLOSUM62 encoding represents an evolutionary approach to protein sequence representation that captures the substitution patterns and evolutionary relationships between amino acids. The BLOSUM matrices were originally developed by Henikoff and Henikoff in 1992 through statistical analysis of highly-conserved protein sequence blocks derived from the BLOCKS database, which contains ungapped alignments of the most conserved regions of protein families. The BLOSUM62 matrix, specifically, was constructed from sequence blocks with no more than 62% sequence identity, striking an optimal balance between capturing evolutionary relationships and maintaining statistical significance. Each element in the BLOSUM62 matrix represents the log-odds ratio of the observed frequency of amino acid substitutions versus the expected frequency based on random chance, with positive values indicating favourable substitutions and negative values representing unfavourable ones. This matrix inherently encodes evolutionary constraints, physicochemical similarities, and functional relationships between amino acids, making it particularly valuable for protein modification site prediction, where evolutionary conservation patterns play crucial roles.

For sequence encoding implementation, each amino acid in the 41-residue peptide was converted to its corresponding 22-dimensional BLOSUM62 vector representation. For example, a lysine (K) residue is represented by the 22-element vector [K→A, K→R, K→N, K→D, K→C, K→Q, K→E, K→G, K→H, K→I, K→L, K→K, K→M, K→F, K→P, K→S, K→T, K→W, K→Y, K→V] with values [−1, 3, 0, −1, −3, 1, 1, −2, −1, −3, −3, 5, −1, −3, −1, 0, −1, −3, −2, −2], where the value 5 at position 12 (K→K) represents the self-substitution score and other values reflect the evolutionary likelihood of substituting lysine with each amino acid. Similarly, an alanine (A) residue would be encoded as [4, −1, −2, −2, 0, −1, −1, 0, −2, −1, −1, −1, −1, −2, −1, 1, 0, −3, −2, 0, 0, 0], demonstrating how each amino acid maintains its unique evolutionary substitution profile within the encoding scheme.

Both padding characters (X) and selenocysteine (U) were assigned zero values across all 20 positions in their BLOSUM62 vectors to indicate undefined evolutionary relationships, while maintaining dimensional consistency. Each 41-residue peptide sequence was transformed into a 41 × 20 feature matrix and flattened to generate 820-dimensional feature vectors that encode both positional and evolutionary information.

### Proposed BiGRU model

#### Theoretical foundations of GRU and BiGRU

The Gated Recurrent Unit (GRU) was introduced by Cho et al. in 2014 as a simplified yet effective alternative to Long Short-Term Memory (LSTM) networks for sequence modeling tasks. Unlike traditional recurrent neural networks that suffer from vanishing gradient problems when processing long sequences, GRU incorporates gating mechanisms to selectively retain or discard information at each time step, enabling to capture long-range dependencies essential for understanding sequential patterns in biological data.

The GRU architecture employs two fundamental gates: the reset gate and the update gate. The reset gate determines how much past information should be forgotten, while the update gate controls the balance between retaining previous hidden states and incorporating new information from the current input. This gating mechanism allows GRU to maintain relevant information across extended sequences while remaining computationally more efficient than LSTM networks, which require three gates (forget, input, and output gates) and separate cell states.

BiGRU extends the standard GRU architecture by processing input sequences in both forward and backward directions, simultaneously. While unidirectional GRU models can only access past contextual information at any given position, BiGRU networks capture both past and future context by maintaining two separate hidden state sequences: one processed from left to right (forward direction) and another from right to left (backward direction). This bidirectional processing is particularly advantageous for protein modification site prediction, where the biological significance of a modification site depends on sequence patterns both upstream and downstream of the target residue.

#### BiGRU architecture and information flow

The BiGRU model processes protein sequences through parallel forward and backward GRU layers, each maintaining independent hidden states that evolve according to the gating mechanisms inherent to GRU networks. Given an input sequence $\:x=({x}_{1},\:{x}_{2},\dots\:.,\:{x}_{t},\dots\:,\:{x}_{T})$, the forward GRU processes the sequence from $\:{x}_{1}$ to $\:{x}_{T}$, generating forward hidden states $\:{h}^{f}=({h}_1^f,\:{h}_2^{f},\:\dots\:.,\:{h}_T^{f})$. In parallel, the backward GRU processes the sequence from $\:{x}_{T}$ to $\:{x}_{1}$, producing backward hidden states $\:{h}^{b}=({h}_{T}^{b},\:{h}_{T-1}^{b},\:\dots\:.,\:{h}_{1}^{b})$. During alignment, however, the backward states are typically re-indexed to correspond to the same positions as the forward states: $\:{h}^{b}=({h}_{1}^{b},\:{h}_{2}^{b},\:\dots\:.,\:{h}_{T}^{b})$.

At each time step $\:t$, the forward hidden state $\:{h}_{t}^{f}$ captures contextual information from the beginning of the sequence up to position $\:t$ ($\:{x}_{1}$ to $\:{x}_{t}$), while the backward hidden state $\:{h}_{t}^{b}$ encodes information from the end of the sequence back to the position $\:t$ ($\:{x}_{T}$ to $\:{x}_{t}$). The final bidirectional representation at position $\:t$ is typically formed by concatenating these two hidden states:

1 $$\:\begin{array}{c}h_t=\left[\:h_t^f;\:h_t^b\right]\end{array}$$

This yields a rich, context-aware representation that incorporates both upstream and downstream information surrounding each amino acid position.

The mathematical formulation of the GRU update equations for each direction follows the standard gating mechanisms:

Reset gate:

2 $$\:\begin{array}{c}r_t=\sigma\:\left(W_rx_t+U_rh_{t-1}+b_r\right)\end{array}$$

Update gate:

3 $$\:\begin{array}{c}z_t=\sigma\:\left(W_zx_t+U_zh_{t-1}+b_z\right)\end{array}$$

Candidate hidden state:

4 $$\:\begin{array}{c}\widetilde{h_t}=\tanh(W_hx_t+U_h\left(r_t\:\odot\:\:h_t\right)+\:b_h)\end{array}$$

Final hidden state:

5 $$\:\begin{array}{c}h_t=\left(1-z_t\right)\odot\:\:h_{t-1}+\:z_t\:\odot\:\:\widetilde{h_t}\end{array}$$

where σ represents the sigmoid activation function, ⊙ denotes element-wise multiplication, $\:W$ and $\:U$ are learnable weight matrices, while $\:b$ represents bias vectors.

#### Proposed BiGKbhb architecture

Based on the comprehensive optimization studies discussed in this paper, we developed BiGKbhb, a specialized deep learning architecture optimized for Kbhb modification site identification. The model architecture integrates the optimal configurations determined through the evaluation of window sizes, encoding strategies, and architectural parameters. The BiGKbhb architecture consists of the following components arranged in a sequential pipeline:

- Input Layer: It accepts BLOSUM62-encoded peptide sequences represented as 2D matrices of shape 41 × 20.
- BiGRU Layer: It is a single layer with 192 units to capture both forward and backward contextual dependencies, enabling integration of information from amino acids upstream and downstream of potential modification sites.
- Batch Normalization: It stabilizes training by normalizing activations, reducing internal covariate shifts, and enabling higher learning rates, while maintaining gradient flow.
- Dropout Regularization: A dropout rate of 0.5 prevents overfitting by randomly setting 50% of neurons to zero during training, encouraging robust feature learning.
- Global Max-pooling in 1D : It extracts the most salient features from BiGRU output by selecting maximum values across the temporal dimension, creating fixed-size representations, while preserving informative features.
- Output Layer: It is a dense layer with sigmoid activation, which produces probability scores in the range [0, 1], indicating the likelihood of Kbhb modification at the central lysine residue. For binary classification, we used a decision boundary threshold of 0.5 to convert these continuous probability scores into discrete class predictions.

The model depends on an Adam optimizer (learning rate = 0.001), and it is trained for 60 epochs with early stopping (patience of 10 epochs) and model checkpointing, using a binary cross-entropy loss function appropriate for distinguishing modified from non-modified lysine residues.

Before model training, all input features derived from each encoding strategy were normalized using MinMax scaling to a [0,1] range. This step was necessary due to the heterogeneous numerical scales of the encoding outputs (e.g., BLOSUM62 scores, physicochemical descriptors, and transformer-based embeddings). To prevent data leakage, scaling was performed independently within each fold of the 10-fold cross-validation. The scaler was fit on the training data and then applied to both the training and validation subsets. For the independent test set, the scaler was fit on the full training set and subsequently applied to the test data.

We evaluated several normalization methods, including standardization and vector normalization, and observed that MinMax scaling consistently yielded marginally improved performance, particularly with our BiGRU-based architecture, which is known to be sensitive to input magnitudes due to its gating mechanisms.

### Comparative deep learning models

DNN represents a traditional feed-forward architecture consisting of multiple fully-connected layers with non-linear activation functions. DNN models process input features through sequential dense layers, learning complex non-linear mappings between BLOSUM62-encoded sequence features and Kbhb modification labels. While DNNs excel at capturing intricate feature interactions and non-linear relationships, they do not inherently account for the sequential nature of protein sequences, treating each position independently without considering positional dependencies or local sequence context that may be crucial for modification site recognition.

A 1DCNN depends on convolution operations with learnable filters to detect local patterns and motifs within protein sequences. The 1DCNN architectures utilize multiple convolutional layers with varying kernel sizes to capture sequence patterns at different scales, followed by pooling operations that reduce dimensionality while preserving the most salient features. This approach is particularly effective for identifying short sequence motifs and local structural patterns that characterize modification sites. The 1DCNNs can capture position-invariant features and are computationally efficient, making them well-suited for detecting conserved sequence signatures that may occur at various positions relative to the modification site.

The LSTM networks represent a specialized recurrent neural network architecture designed to address the vanishing gradient problem inherent in traditional RNNs, when processing long sequences. They employ a sophisticated gating mechanism that consists of forget gates, input gates, and output gates, along with separate cell states that enable the selective retention and forgetting of information over extended sequences. This architecture allows LSTMs to capture long-range dependencies in protein sequences, making them particularly valuable for identifying spatially separated sequence patterns that influence modification site recognition. The cell state mechanism enables LSTMs to maintain relevant information across many time steps, while selectively updating or discarding less relevant information.

The BiLSTM extends the standard LSTM architecture by processing input sequences in both forward and backward directions, simultaneously, similar to the BiGRU approach but with a more complex LSTM gating mechanism. BiLSTM networks maintain two separate hidden state sequences: forward states that process sequences from the N-terminus to the C-terminus and backward states that process sequences in the reverse direction. This bidirectional processing enables comprehensive capturing of both past and future contextual information at each amino acid position, providing a complete sequence context essential for accurate modification site prediction. The combination of the LSTM sophisticated memory management with bidirectional processing makes BiLSTM particularly powerful for tasks requiring an understanding of both local and distant sequence dependencies in protein modification recognition.

### Evaluation metrics

Accurate evaluation of protein PTM prediction models requires rigorous assessment frameworks that capture multiple dimensions of classification performance. Although our datasets were balanced, a comprehensive evaluation remains essential due to the biological significance of different prediction errors in downstream proteomics applications. False negatives (missed modification sites) and false positives (incorrectly predicted sites) have distinct implications for biological interpretation and experimental validation strategies, necessitating multiple complementary evaluation metrics that assess different aspects of model performance.

The evaluation framework employed in this study addresses these challenges through multiple assessment strategies: (1) cross-validation to evaluate model learning capability and internal consistency, (2) independent test set evaluation to assess generalization to unseen data, (3) multiple performance metrics to capture different aspects of classification performance, and (4) statistical significance testing to ensure robust comparative analysis between different methodologies.

Model performance was evaluated using six complementary metrics calculated from the confusion matrix elements: true positives ($\:TP$), true negatives ($\:TN$), false positives ($\:FP$), and false negatives ($\:FN$).

- Accuracy (ACC) measures the overall proportion of correctly-classified samples across both positive and negative classes.

6 $$\:\begin{array}{c}ACC=\frac{TP+\:TN}{TP+\:TN+\:FP+\:FN}\end{array}$$

- Recall (RC), also known as sensitivity, quantifies the model ability to correctly identify positive Kbhb modification sites.

7 $$\:\begin{array}{c}RC=\:\frac{TP}{TP+\:FN}\end{array}$$

- Precision (PR), also referred to as positive predictive value, measures the proportion of predicted positive sites that are modified.

8 $$\:\begin{array}{c}PR=\:\frac{TP}{TP+\:FP}\end{array}$$

- F1 score provides the harmonic mean of precision and recall, offering a balanced assessment that accounts for both false positives and false negatives.

9 $$\:\begin{array}{c}F1=2\:\times\:\:\frac{PR\:\times\:\:RC}{PR+RC}\end{array}$$

- Matthews Correlation Coefficient (MCC) serves as a balanced metric that accounts for all four confusion matrix elements, providing a comprehensive assessment of prediction quality that considers both positive and negative class prediction performance.

10 $$\:\begin{array}{c}MCC=\frac{TP\:\times\:\:TN-FP\:\times\:\:FN}{\sqrt{\left(TP+FP\right)\left(TP+FN\right)\left(TN+FP\right)\left(TN+FN\right)}}\end{array}$$

- Area Under the Receiver Operating Characteristic (ROC) curve (AUC) measures the model discriminative ability across all classification thresholds by plotting the true positive rate against the false positive rate.

11 $$AUC=\int_0^1TPR\left(FPR^{-1}\left(x\right)\right)dx$$

where TPR is the True Positive Rate and FPR is the False Positive Rate. AUC values range from 0.5 (random classification) to 1.0 (perfect classification).

For *k*-fold cross-validation results, performance metrics were reported as mean ± standard deviation across all folds to capture both central tendency and variability.

12 $$\:\begin{array}{c}\overline X=\:\frac1K\:\sum\:_{i=1}^kX_i\end{array}$$

13 $$\:\begin{array}{c}SD=\:\sqrt{\frac1{K-1}\sum\:_{i=1}^k\left(X_i-\:\overline X\right)^2}\end{array}$$

where ${X}_{i}$ represents the metric value for fold $i$, $\stackrel{-}{X}$ is the mean performance across folds, and $SD$ is the standard deviation indicating performance consistency across different data partitions.

The statistical significance of performance differences between models was assessed using DeLong test, a non-parametric method specifically designed for comparing AUC values from correlated ROC curves. This test is particularly appropriate for comparing models evaluated on the same dataset, as it accounts for the correlation structure inherent in paired predictions.

DeLong test computes the difference in AUC values and their variance using the structural components of the ROC curves.

14 $$\:\begin{array}{c}Z=\:\frac{{AUC}_1-\:{AUC}_2}{\sqrt{var\:\left({AUC}_1-\:{AUC}_2\right)}}\end{array}$$

The variance term accounts for the covariance between the two AUC estimates. Under the null hypothesis of equal AUC values, the test statistic attribute follows a standard normal distribution.

To control for the family-wise error rate when conducting multiple pairwise comparisons, the Bonferroni correction was applied by adjusting individual *P*-values.

15 $$\:\begin{array}{c}P_{corrected}=\:P_{row}\:\times\:\:n_{comparisons}\end{array}$$

where ${n}_{comparisons}$ represents the total number of statistical tests performed. This conservative correction ensures that the probability of making at least one Type I error across all comparisons remains at or below the specified significance level (α = 0.05).

ROC curves provide a comprehensive visualization of model performance across all possible classification thresholds by plotting the true positive rate against the false positive rate, offering threshold-independent performance assessment, visual comparison of multiple models, identification of optimal operating points, and comprehensive evaluation of discriminative capability [70]. In this study, ROC curves were generated for both cross-validation and independent test set evaluations, where cross-validation curves were averaged using vertical averaging at fixed false positive rate values with confidence intervals to indicate variability across folds, while independent test set ROC curves provided unbiased estimates of model generalization performance on completely unseen data.

## Results

### Comparative analysis of position-specific amino acid preferences at Kbhb sites in human, mouse, and fungal proteomes

To understand local sequence environments influencing Kbhb site recognition, we performed motif analysis on human, mouse, and fungal datasets. Sequence patterns within a window size of 41 amino acids were examined using Two-sample logo analysis [71]. This approach identifies the statistically-significant differences in amino acid frequencies between Kbhb-modified and non-modified lysine sites. In these logos, residues displayed in the upper portion (enriched) represent amino acids that occur significantly more frequently at specific positions in Kbhb-modified sites compared to non-modified lysine sites. Conversely, residues in the lower portion (depleted) represent amino acids that occur with significantly lower frequency in Kbhb-modified sites. The percentage values at the *y*-axis indicate the maximum frequency difference between Kbhb and non-Kbhb sites at any position, quantifying the strength of the observed motif patterns.

**Fig. 2:**
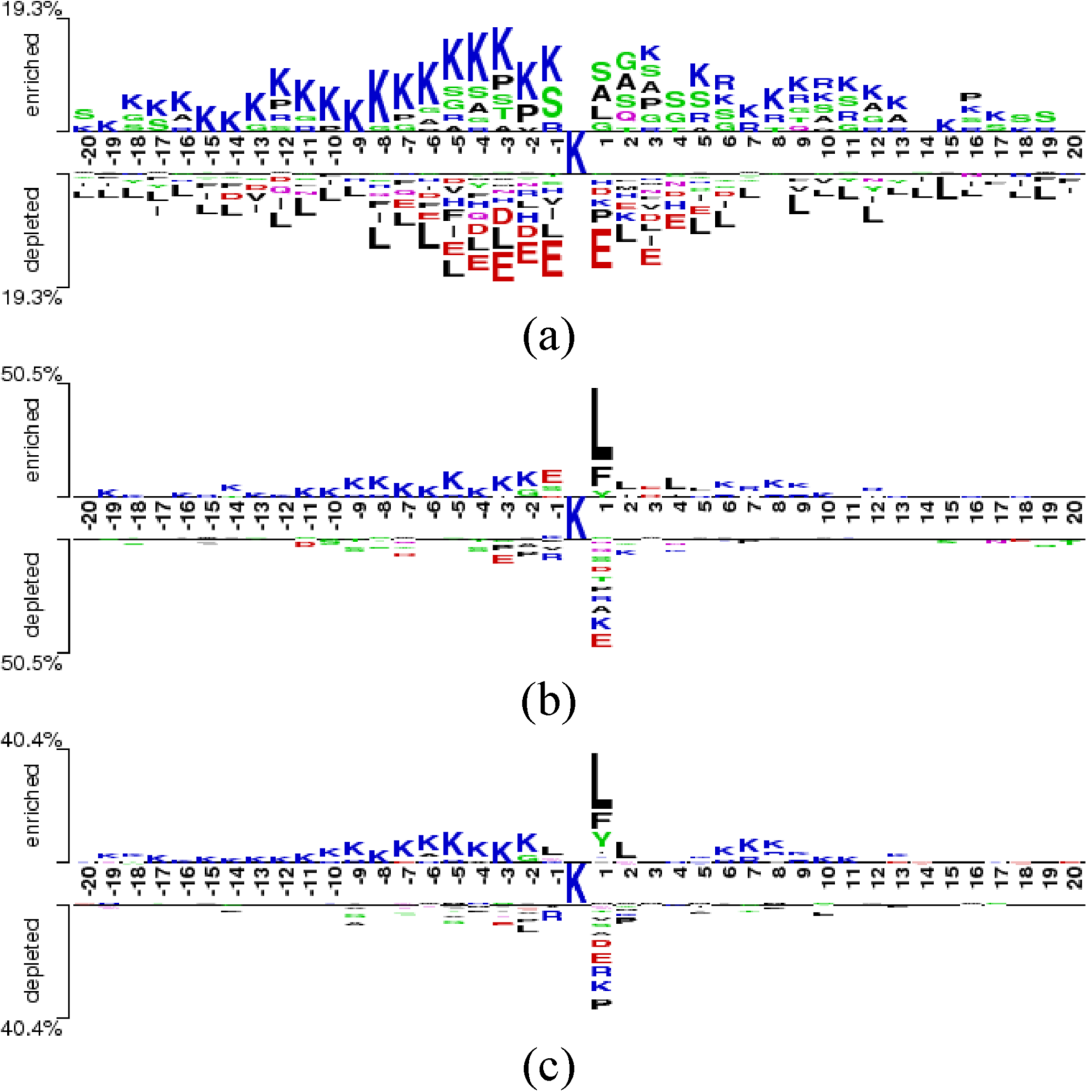
Position-specific amino acid preferences surrounding Kbhb sites in human, mouse, and fungal (*U. virens*) proteins determined by two-sample logo analysis with significantly enriched or depleted residues identified using Student’s *t*-test with Bonferroni correction (*P* < 0.05)

The human Kbhb site two-sample logo (Fig. 2a) shows amino acid preferences. The upstream region (positions − 20 to −1) shows significant enrichment of positively-charged lysine (K) residues, showing an electrostatic mechanism influencing substrate recognition. The downstream region exhibits a more diverse pattern with enrichment of small residues, including serine (S), alanine (A), and glycine (G) at positions + 1 to + 6, potentially providing structural flexibility that facilitates enzymatic access to the modification site. Notably, the logo shows consistent depletion of hydrophobic leucine (L) residues and negatively-charged glutamic acid (E) at positions immediately preceding the Kbhb site.

Analysis of the mouse proteome shows a stronger sequence signature around Kbhb sites compared to human proteins (Fig. 2b). Immediately downstream of the modification site, a striking enrichment of both phenylalanine (F) and L at position + 1 dominates the motif, ensuring that these hydrophobic residues play critical roles in mouse Kbhb site recognition. Proximal to the modification site, the upstream region displays a notable distribution of positively-charged (K) residues at different positions, creating an electropositive environment that may facilitate enzyme-substrate interactions. Interestingly, glutamic acid (E) appears enriched at position − 1, contrasting with its depletion in the human motif. The downstream region beyond position + 1 shows relatively-sparse enrichment patterns, with occasional lysine residues appearing at distal positions. In the depleted portion, the under-representation of various residues is observed.

Examination of fungal Kbhb sites from *U. virens* reveals characteristic sequence preferences with a substantial 40.4% maximum frequency difference between Kbhb and non-Kbhb (Fig. 2c). Most prominently, L and F are strongly enriched at position + 1, confirming the pattern observed in the mouse dataset but contrasting with human preferences. Further downstream, another lysine enrichment appears at positions + 6 to + 8, showing a potential structural periodicity that may influence modification site recognition. The upstream flanking region exhibits an accumulation of positively-charged (K) residues at all positions. Among depleted residues, the most notable pattern is the significant underrepresentation of lysine at position 0 (the modification site itself), along with negatively charged residues (D, E) and proline (P) at nearby positions.

The comparative analysis of three proteomes highlights both conserved elements and significant variations. A striking difference is observed in the strength of the sequence motifs, with maximum frequency differences of 19.3%, 50.5%, and 40.4% for human, mouse, and fungal datasets, respectively, signifying varying degrees of sequence specificity across species. While all three species show enrichment of positively-charged lysine residues in upstream regions (a conserved feature), they differ markedly at position + 1: humans prefer small, flexible residues (S, A, G), whereas mouse and fungal proteins favour hydrophobic residues (F, L). These species-specific sequence preferences likely reflect evolutionary adaptations to different cellular environments.

The significant differences observed between the sequence logo plots of Kbhb-modified and non-modified sites arise from several factors, including enzyme-specific substrate recognition preferences (e.g., CBP/p300 acetyltransferases that also catalyze β-hydroxybutyrylation), local structural accessibility, and evolutionary conservation of functionally-important motifs. The enrichment or depletion of specific amino acids around the modified lysine residues reveals selective pressures and functional constraints associated with Kbhb modification. Such distinct sequence signatures are precisely what enable computational models like BiGKbhb to effectively distinguish between modified and non-modified sites, underscoring their importance in predictive modeling.

### Optimization of sequence context for enhanced Kbhb site prediction

The prediction performance of deep learning models for PTM sites is highly dependent on the sequence context provided, necessitating careful optimization of the window size surrounding the central lysine residue. To identify the optimal peptide length for Kbhb site prediction, we systematically evaluated nine different window sizes ranging from 15 to 51 amino acids. This range encompasses the window sizes used in existing Kbhb predictors: KbhbXG has 15 residues while SLAM has 51 residues, alongside our core evaluation range of 35–47 amino acids (corresponding to ± 17 to ± 23 residues flanking the central lysine).

For each window size, we optimized the BiGRU model architecture using the Python Keras Tuner [72] to ensure fair comparison between different context lengths. Supplementary Table **S1** presents the optimized architectural parameters for each window size configuration. Notably, all optimized models maintain a single BiGRU layer with the number of units varying from 64 to 256 depending on the window size. While larger window sizes (41–47) generally require more complex architectures with 192 units and higher dropout rates (0.4–0.5), smaller window sizes (35–39) perform optimally with fewer units and lower dropout rates. This pattern shows that as the sequence context expands, more complex architectures are necessary to effectively capture the increased information content without overfitting.

The performance of each window size was evaluated on the human dataset using both 10-fold cross-validation and an independent test set, with results presented in Table 4. The cross-validation results demonstrate consistently-high performance across all window sizes, with accuracy ranging from 0.835 to 0.845 and AUC values between 0.914 and 0.919. However, the test set results reveal more pronounced differences, indicating varying degrees of generalization capability.

**Table 4:** Comparative performance metrics of different window sizes (WS) on the human Kbhb dataset. Values represent mean ± SD across folds. Boldface values indicate the best performance for each metric

**WS**	**10-fold cross-validation**				**Test set**			
	**ACC**	**F1**	**MCC**	**AUC**	**ACC**	**F1**	**MCC**	**AUC**
51	0.831 ± 0.022	0.834 ± 0.019	0.662 ± 0.044	0.909 ± 0.018	0.850	0.853	0.701	0.916
47	0.839 ± 0.018	0.843± 0.018	0.680 ± 0.036	0.919 ± 0.016	0.822	0.822	0.646	0.922
45	0.835 ± 0.016	0.836± 0.020	0.673 ± 0.032	0.917 ± 0.012	0.803	0.817	0.606	0.911
43	0.845 ± 0.017	0.846± 0.017	0.690 ± 0.033	0.920 ± 0.011	0.810	0.821	0.619	0.899
41	**0.840 ± 0.013**	**0.841± 0.014**	**0.680 ± 0.026**	**0.923 ± 0.012**	**0.824**	**0.833**	**0.648**	**0.920**
39	0.837 ± 0.014	0.840± 0.014	0.678 ± 0.029	0.915 ± 0.011	0.820	0.832	0.639	0.914
37	0.836 ± 0.016	0.839± 0.017	0.674 ± 0.032	0.917 ± 0.012	0.815	0.829	0.630	0.914
35	0.839 ± 0.016	0.843± 0.016	0.680 ± 0.033	0.919 ± 0.011	0.798	0.799	0.598	0.902
15	0.823 ± 0.025	0.825 ± 0.026	0.647 ± 0.050	0.907± 0.018	0.801	0.823	0.590	0.908

Among all configurations, the window size of 41 (± 20 residues) achieved the most balanced performance, with optimal generalization between training and testing. This window size achieved test set metrics including ACC of 0.824, F1-score of 0.833, MCC of 0.648, and AUC of 0.920. While some larger window sizes achieved marginally higher cross-validation performance, they showed signs of overfitting with degraded test set metrics. Conversely, smaller window sizes demonstrated insufficient context capture that is particularly evident in the lower precision and AUC values on the test set.

**Fig. 3:**
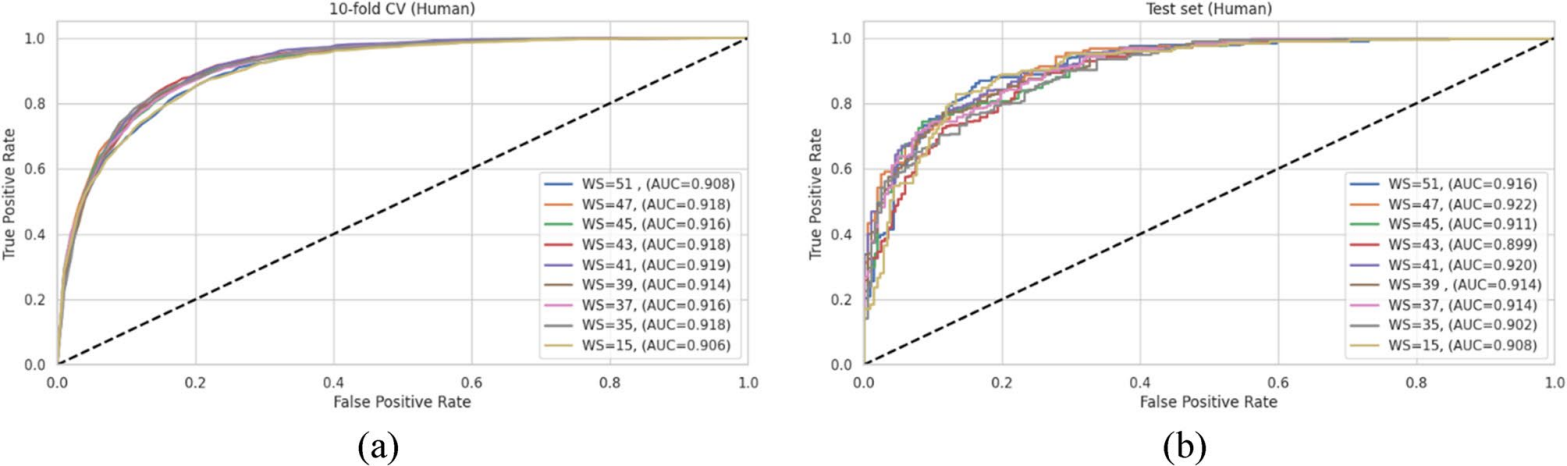
ROC curves illustrating the classification performance of BiGRU models with different window sizes on (**a**) a 10-fold cross-validation and (**b**) an independent test set of the human dataset

The ROC curves presented in Fig. 3 provide further visual confirmation of the window-size-based performance comparison. The curves for window size 41 maintain high AUC values with consistent performance between cross-validation and test set results, indicating robust generalization capability.

Based on this comprehensive analysis, we selected a window size of 41 for all subsequent experiments in our proposed model development. This selection represents an optimal balance between capturing sufficient sequence context for accurate prediction, while avoiding diminishing returns and potential overfitting associated with excessively large window sizes.

### Comprehensive evaluation of protein sequence encoding strategies for Kbhb site prediction

The choice of protein sequence representation fundamentally influences machine learning model performance in PTM prediction tasks. To identify the optimal encoding strategy for Kbh site prediction, we systematically evaluated seven distinct feature representation methods spanning four major categories: embedding-based approaches (ESM, ProtBERT, ProtGPT2), sequence context-based encoding (one-hot), physicochemical properties-based methods (CTD, AAP), and evolutionary representation (BLOSUM62).

For each encoding method, we optimized the BiGRU model architecture using the Python Keras Tuner library to ensure fair comparison across different feature representations. Table 5 presents the optimized architectural parameters, revealing interesting patterns in model complexity requirements. Embedding-based methods (ESM, ProtBERT, ProtGPT2) require varying architectural complexity, with ESM demanding the highest capacity (224 units), while ProtBERT and ProtGPT2 perform optimally with moderate complexity (128 units). Traditional encoding methods show diverse requirements: BLOSUM62 requires substantial capacity (192 units with high dropout), while simpler methods like one-hot, CTD, and AAP achieved optimal performance with fewer parameters (64–69 units). This pattern confirms that the information richness of different encoding schemes directly influences the architectural complexity needed for effective feature extraction.

**Table 5:** Optimized BiGRU model architectural parameters for different protein sequence encoding methods in Kbhb site prediction

Encoding method	ProtBERT	ProtGPT2	ESM	Onehot	CTD	AAP	BLOSUM
Number of BiGRU layers	1	1	1	1	1	1	1
Number of units/layers	128	128	224	64	69	64	192
Batch normalization	✔	✔	✔	✔	✔	✔	✔
Activation function	ReLU	ReLU	ReLU	ReLU	ReLU	ReLU	ReLU
Dropout/layer	0.4	0.1	0.4	0.5	0.1	0.1	0.5
Global max pooling 1d	✔	✔	✔	✔	✔	✔	✔
Learning rate	0.001	0.0001	0.001	0.001	0.01	0.0001	0.001
Number of epochs	60	60	60	60	80	80	60

The comparative evaluation of the human dataset (Table 6) reveals striking performance differences across encoding strategies. BLOSUM62 achieved the highest performance with cross-validation with ACC of 0.839, RC of 0.853, PR of 0.830, F1-score of 0.841, MCC of 0.680, and AUC of 0.919. Remarkably, the test set performance (ACC of 0.824, and AUC of 0.920) demonstrated excellent generalization capability. One-hot encoding emerged as the second-best performer, achieving competitive metrics with cross-validation ACC (CV-ACC) of 0.786 and a test ACC of 0.791.

**Table 6:** Comparative performance evaluation of protein sequence encoding methods for Kbhb site prediction on the human dataset. Boldface values indicate the best performance for each metric

Encoding method	10-fold cross-validation				
	ACC	RC	PR	MCC	AUC
ProtBERT	0.743 ± 0.022	0.739 ± 0.033	0.744 ± 0.023	0.487 ± 0.043	0.815 ± 0.023
ProtGPT2	0.759 ± 0.016	0.750 ± 0.028	0.763 ± 0.024	0.518 ± 0.031	0.833 ± 0.020
ESM	0.754 ± 0.023	0.753 ± 0.029	0.754 ± 0.031	0.509 ± 0.045	0.839 ± 0.020
Onehot	0.786 ± 0.028	0.827 ± 0.047	0.764 ± 0.030	0.575 ± 0.057	0.866 ± 0.020
CTD	0.723 ± 0.033	0.835 ± 0.068	0.687 ± 0.049	0.466 ± 0.046	0.794 ± 0.026
AAP	0.753 ± 0.026	0.750 ± 0.036	0.753 ± 0.026	0.506 ± 0.051	0.826 ± 0.018
**BLOSUM**	**0.840 ± 0.013**	**0.853 ± 0.028**	**0.830 ± 0.020**	**0.680 ± 0.026**	**0.923 ± 0.013**
	Test set				
ProtBERT	0.741	0.753	0.750	0.481	0.813
ProtGPT2	0.774	0.799	0.774	0.548	0.838
ESM	0.760	0.836	0.738	0.522	0.814
Onehot	0.791	0.854	0.770	0.583	0.868
CTD	0.763	0.813	0.751	0.525	0.828
AAP	0.760	0.808	0.750	0.520	0.839
**BLOSUM**	**0.824**	**0.845**	**0.822**	**0.648**	**0.920**

Among embedding-based methods, ProtGPT2 showed the best performance with CV-ACC of 0.759 and test accuracy of 0.774, followed by ESM and ProtBERT. Interestingly, the sophisticated pre-trained embeddings underperformed compared to traditional encoding methods.

The mouse dataset results shown in Table 7 confirmed the performance of BLOSUM62 encoding, which achieved high cross-validation metrics (ACC of 0.835, RC of 0.868, and AUC of 0.918) and strong test set generalization (ACC of 0.832, RC of 0.902, and AUC of 0.902). The AAP method demonstrated notably better performance on the mouse dataset compared to humans, ranking second with cross-validation and test ACC of 0.779 and 0.728, respectively. This species-specific improvement likely reflects the influence of the smaller mouse dataset (1,244 versus 4,210 human samples), where AAP intermediate-complexity physicochemical representation may achieve an optimal bias-variance trade-off compared to the more complex encoding strategies.

**Table 7:** Comparative performance evaluation of protein sequence encoding methods for Kbhb site prediction on the mouse dataset. Boldface values indicate the best performance for each metric

Encoding method	10-fold cross-validation				
	ACC	RC	PR	MCC	AUC
ProtBERT	0.682 ± 0.051	0.750 ± 0.084	0.661 ± 0.047	0.370 ± 0.104	0.747 ± 0.052
ProtGPT2	0.708 ± 0.023	0.697 ± 0.036	0.715 ± 0.034	0.417 ± 0.047	0.780 ± 0.033
ESM	0.705 ± 0.027	0.742 ± 0.054	0.698 ± 0.046	0.415 ± 0.049	0.776 ± 0.042
Onehot	0.751 ± 0.046	0.795 ± 0.052	0.733 ± 0.051	0.505 ± 0.092	0.812 ± 0.046
CTD	0.702 ± 0.028	0.791 ± 0.057	0.673 ± 0.030	0.412 ± 0.057	0.762 ± 0.039
AAP	0.779 ± 0.037	0.818 ± 0.032	0.763 ± 0.053	0.562 ± 0.072	0.851 ± 0.038
**BLOSUM**	**0.835 ± 0.029**	**0.868 ± 0.039**	**0.815 ± 0.033**	**0.672 ± 0.059**	**0.919 ± 0.018**
	Test set				
ProtBERT	0.672	0.574	0.700	0.346	0.701
ProtGPT2	0.696	0.639	0.709	0.392	0.756
ESM	0.608	0.525	0.615	0.215	0.723
Onehot	0.704	0.705	0.694	0.408	0.809
CTD	0.704	0.771	0.671	0.414	0.757
AAP	0.728	0.738	0.714	0.456	0.792
**BLOSUM**	**0.832**	**0.902**	**0.786**	**0.672**	**0.902**

The fungal dataset exhibited the most dramatic performance differences as shown in Table 8, with BLOSUM62 achieving outstanding results (CV-ACC of 0.869, test ACC of 0.871, and AUC of 0.944 and 0.945, respectively). This represents the highest performance observed across all species and encoding combinations. One-hot encoding again ranked second (CV-ACC of 0.782, and test ACC of 0.797), while AAP showed moderate performance as the third-best method. The embedding-based methods showed consistently poor performance on the fungal dataset, with ProtBERT achieving the lowest scores with a CV-ACC of 0.653 and a test ACC of 0.669.

**Table 8:** Comparative performance evaluation of protein sequence encoding methods for Kbhb site prediction on the fungal dataset. Boldface values indicate the best performance for each metric

Encoding method	10-fold cross-validation				
	ACC	RC	PR	MCC	AUC
ProtBERT	0.653 ± 0.031	0.660 ± 0.060	0.650 ± 0.034	0.307 ± 0.063	0.711 ± 0.029
ProtGPT2	0.676 ± 0.032	0.661 ± 0.047	0.680 ± 0.036	0.353 ± 0.063	0.737 ± 0.032
ESM	0.686 ± 0.013	0.727 ± 0.046	0.672 ± 0.023	0.376 ± 0.023	0.756 ± 0.015
Onehot	0.782 ± 0.023	0.835 ± 0.037	0.756 ± 0.033	0.569 ± 0.044	0.869 ± 0.024
CTD	0.690 ± 0.020	0.742 ± 0.048	0.671 ± 0.025	0.383 ± 0.039	0.760 ± 0.021
AAP	0.744 ± 0.030	0.731 ± 0.053	0.749 ± 0.033	0.489 ± 0.060	0.828 ± 0.033
**BLOSUM**	**0.869 ± 0.021**	**0.875 ± 0.045**	**0.864 ± 0.015**	**0.740 ± 0.043**	**0.945 ± 0.015**
	Test set				
ProtBERT	0.669	0.642	0.698	0.340	0.711
ProtGPT2	0.688	0.716	0.695	0.375	0.731
ESM	0.717	0.735	0.726	0.433	0.773
Onehot	0.797	0.809	0.804	0.594	0.888
CTD	0.707	0.803	0.688	0.416	0.779
AAP	0.730	0.679	0.775	0.466	0.803
**BLOSUM**	**0.871**	**0.877**	**0.877**	**0.742**	**0.945**

**Fig. 4:**
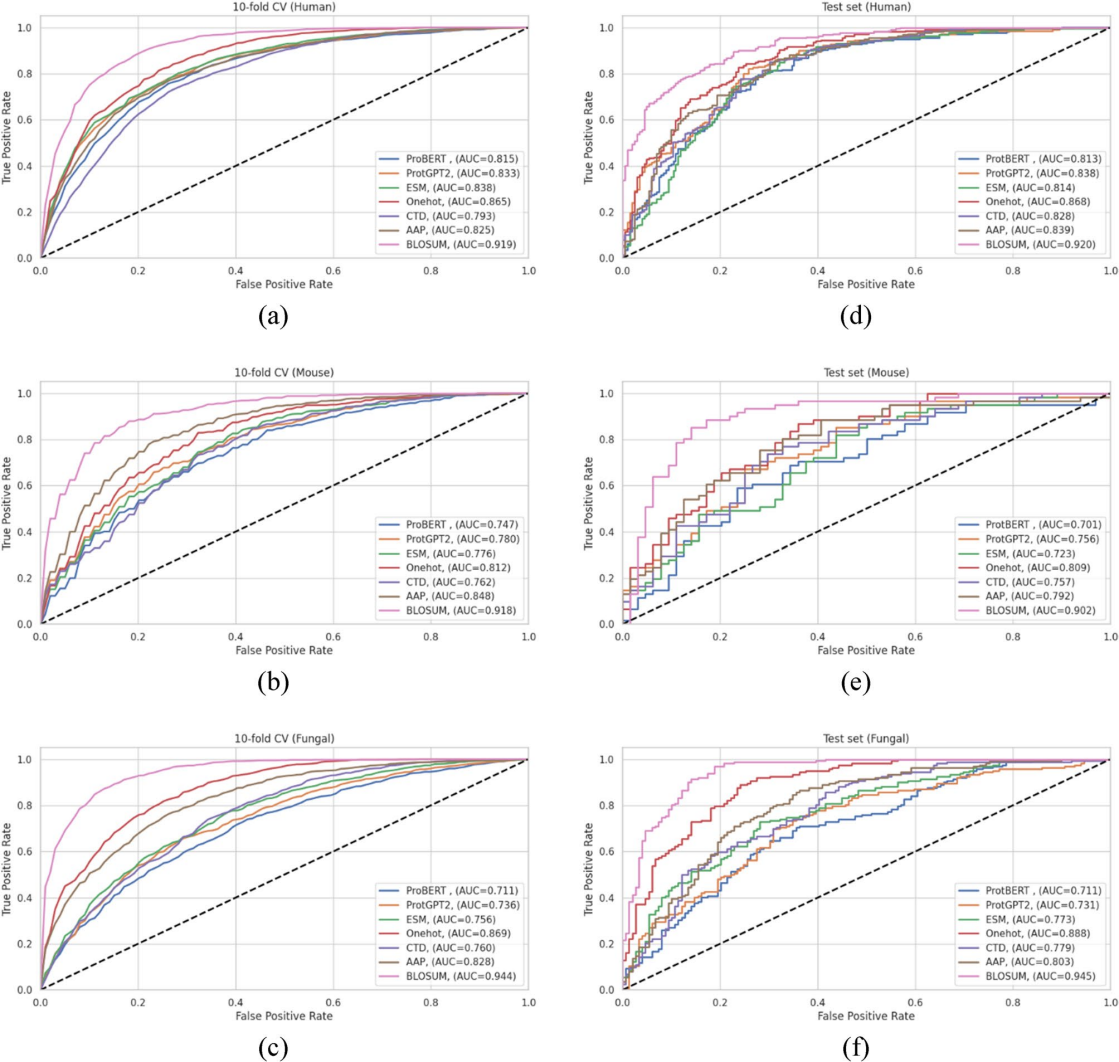
ROC curves comparing protein sequence encoding methods across three species: (**a**-**c**) 10-fold CV performance; (**d**-**f**) independent test set performance for human, mouse, and fungal datasets, respectively

The ROC curves presented in Fig. 4 provide a comprehensive visualization of encoding method performance across all three species. BLOSUM62 consistently maintains the highest AUC values with consistency between cross-validation and test sets across all species. This performance of BLOSUM can be attributed to the incorporation of evolutionary substitution patterns that directly reflect the functional constraints governing protein sequences. Based on these comprehensive evaluations, BLOSUM62 was selected as our proposed protein sequence encoding strategy and designated the proposed BiGRU model, which will be employed in all subsequent analyses and comparisons.

It is important to acknowledge a methodological limitation concerning the application of pre-trained protein language models in this study. The transformer-based embeddings (ESM2, ProtBERT, ProtGPT2) were originally trained on full-length protein sequences to capture global structural context and long-range dependencies. In contrast, our approach involves applying these models to fixed-length 41-residue peptide windows centred on the lysine site of interest. This contextual mismatch may have led to suboptimal utilization of their representational capacity and contributed to the comparatively weaker performance of embedding-based methods.

In contrast, the BLOSUM62 encoding, which captures local evolutionary substitution patterns, is inherently better suited for residue-centred sequence windows. The better performance of BLOSUM62 observed in our comparative analysis may therefore reflect its natural compatibility with local-window-based PTM prediction, whereas transformer-based embeddings may require incorporation of full-sequence context or task-specific adaptation to reach their full potential.

### Comparative evaluation of deep learning architectures for Kbhb site prediction

Having established BLOSUM as the optimal encoding strategy, we conducted a comprehensive comparison of different deep learning architectures to identify the most suitable model type for Kbhb site prediction. This evaluation was essential to determine whether the sequential nature of protein sequences and the positional dependencies of modification sites favour specific neural network architectures over others. We systematically compared six distinct deep learning approaches: DNN, 1DCNN, LSTM, BiLSTM, GRU, and BiGRU.

Each model architecture was optimized using Keras Tuner with BLOSUM62-encoded peptide sequences to ensure fair comparison across different approaches. To prevent overfitting and ensure optimal training, early stopping with patience of 10 epochs and model checkpointing was implemented for all models, with a consistent batch size of 64 across all architectures.

Table S2 presents the optimized architectural parameters for each model type, revealing distinct complexity and training requirements. The 1DCNN architecture has the most complex design with two convolutional layers, multiple kernel sizes (7 and 3), max pooling operations, and five additional dense layers with varying units (192, 352, 224, 224, 128). In contrast, recurrent models demonstrated more streamlined architectures, with LSTM requiring a single layer of 480 units, while BiLSTM and BiGRU achieved optimal performance with fewer units (160 for BiLSTM, 192 for BiGRU), ensuring that bidirectional processing enables more efficient parameter utilization.

Notably, the optimized number of training epochs varies notably across architectures, reflecting differences in learning capacity and convergence behaviour. DNN, 1DCNN, and BiGRU require 60 epochs, whereas LSTM and BiLSTM need 100 and 80 epochs, respectively. Convergence patterns further highlighted these differences: DNN and 1DCNN converged within 15–20 epochs, BiGRU within 20–25 epochs, BiLSTM within 42–50 epochs, and LSTM within 80–90 epochs. These results reveal that bidirectional architectures, particularly BiGRU, achieve efficient learning by balancing model complexity and training stability.

The comparative evaluation on the human dataset reveals clear performance hierarchies among different architectural approaches, as shown in Table S3. BiGRU achieved the best performance with CV-ACC of 0.839, RC of 0.853, PR of 0.830, MCC of 0.680, and AUC of 0.919. Remarkably, the test set performance (ACC of 0.824, and AUC of 0.920) showed good generalization with minimal performance degradation, indicating high model stability.

BiLSTM emerged as the second-best performer with competitive cross-validation metrics with an ACC of 0.843 and an AUC of 0.917, though it showed a more significant performance decline on the test set with an ACC of 0.812 and an AUC of 0.894, revealing potential overfitting tendencies. The unidirectional GRU ranked third with balanced performance across both evaluation sets (CV-ACC of 0.837, test ACC of 0.831), while LSTM showed moderate performance with higher variance between training and testing phases.

Traditional architectures demonstrated inferior performance, with 1DCNN achieving moderate results (CV-ACC of 0.769 and a test ACC of 0.772) and DNN showing the poorest performance (CV-ACC of 0.724, and test ACC of 0.734). These results indicate that the sequential and positional nature of protein modification sites strongly favours recurrent architectures over feed-forward approaches.

The mouse dataset results shown in Table S4 confirm the superiority of bidirectional recurrent architectures, with BiGRU and BiLSTM achieving nearly identical performance levels. BiGRU maintained its leading position with cross-validation ACC and AUC of 0.835 and 0.918, respectively, while demonstrating exceptional test set performance with ACC of 0.832, RC of 0.902, and AUC of 0.902.

Interestingly, the mouse dataset revealed more pronounced performance differences between bidirectional and unidirectional models. While BiLSTM achieved comparable cross-validation performance with an ACC of 0.839 and an AUC of 0.917, the unidirectional GRU showed significant performance degradation on the test set, with an ACC of 0.760 versus 0.813 with cross-validation. This degradation may be attributed to the limited number of samples in the mouse dataset, which could hinder the ability of simpler unidirectional models to generalize, effectively.

The pattern of traditional architectures performing poorly persisted, with DNN and 1DCNN showing limited effectiveness with test ACCs of 0.744 and 0.720, respectively. This consistent trend across species reinforces the importance of capturing sequential dependencies in protein modification prediction tasks.

As shown in Table S5, the fungal dataset revealed the highest overall performance levels, with BiGRU achieving outstanding results across all metrics with CV-ACC of 0.869, test ACC of 0.871, and AUC of 0.944 and 0.945, respectively. This represents the highest performance observed across all species and architectural combinations, revealing that the fungal dataset may possess more distinctive sequence patterns that facilitate accurate prediction.

Notably, the fungal dataset demonstrated exceptional performance convergence among top-performing models, with GRU achieving remarkably similar results to those of BiGRU with CV-ACC of 0.863, test ACC of 0.871, and test AUC of 0.933. This convergence shows that the fungal sequence patterns are sufficiently distinctive that even unidirectional processing can achieve near-optimal performance. LSTM also showed impressive performance on the fungal dataset with test ACC of 0.862, and test AUC of 0.943, confirming that recurrent architectures are particularly well-suited for capturing the complex sequence dependencies in fungal Kbhb sites.

**Fig. 5:**
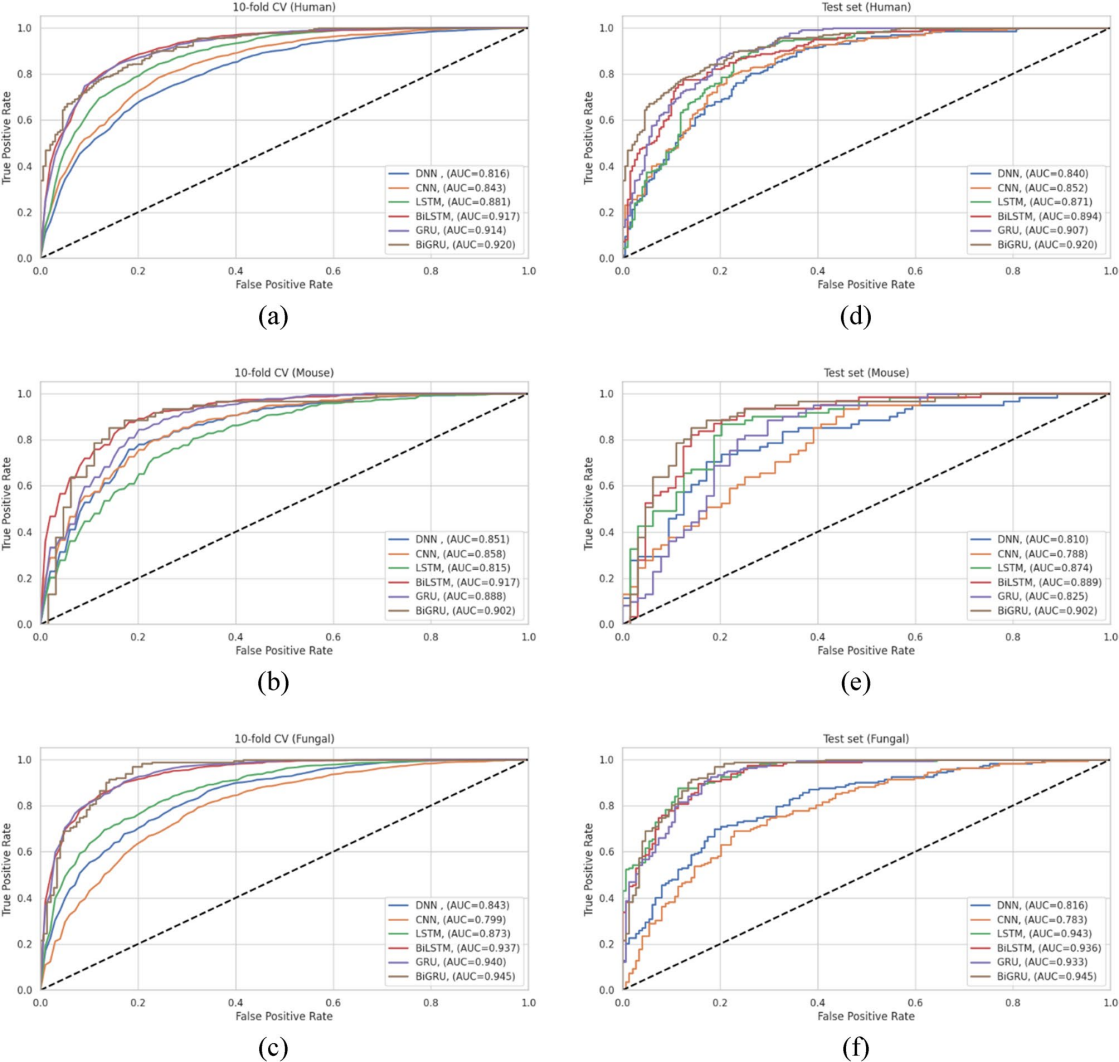
ROC curves comparing deep learning architectures optimized for Kbhb site identification across three species: (**a**-**c**) 10-fold cross-validation; and (**d**-**f**) independent test set performance for the human, mouse, and fungal datasets, respectively

The ROC curves presented in Fig. 5 comprehensively validate the architectural performance patterns observed across the three species. The BiGRU consistently maintains the highest AUC values, confirming its generalization capability and robustness. Based on this comparison, the BiGRU emerged as the optimal choice for Kbhb site prediction across all three species. Consequently, our final model was designated as BiGKbhb. It will be employed in all subsequent comparative analyses.

### Benchmarking BiGKbhb against traditional machine learning classifiers

To comprehensively evaluate the performance of our proposed BiGKbhb model, we conducted extensive comparisons with six established machine learning classifiers: K-Nearest Neighbors (KNN) [73], Support Vector Machines (SVM) [74], Random Forest (RF) [75], Extreme Gradient Boosting (XGBoost) [76], Light Gradient Boosting Machine (LightGBM) [77], and Categorical Boosting (CatBoost) [78]. All classifiers were optimized using GridSearchCV with 10-fold cross-validation to ensure fair comparison with our deep learning approach.

The optimal hyperparameters identified for each classifier were as follows; KNN with 7 neighbors, Manhattan distance metric, and uniform weights; SVM employing RBF kernel with degree = 2 and gamma = auto; RF with 400 estimators and entropy criterion; XGBoost configured with 300 estimators and learning rate = 0.1; LightGBM optimized with 391 estimators, learning rate = 0.01, 52 leaves, and regularization parameters (alpha and lambda) set to 0; and CatBoost trained for 320 iterations. All models utilized the same BLOSUM62-encoded features to ensure consistent input representation across different algorithmic approaches.

As shown in Table S6, the comparative evaluation on the human dataset show Ó that BiGKbhb outperforms other methods across all evaluation metrics. BiGKbhb achieved the highest cross-validation performance with ACC of 0.839, RC of 0.853, PR of 0.830, MCC of 0.680, and AUC of 0.919, while maintaining excellent generalization on the test set with ACC of 0.824, RC of 0.845, PR of 0.833, MCC of 0.648, and AUC of 0.920. Among traditional classifiers, CatBoost emerged as the strongest competitor with cross-validation metrics of ACC of 0.793 and AUC of 0.867.

LightGBM ranked third with competitive performance with a CV-ACC of 0.785 and test ACC of 0.808, followed by XGBoost with a CV-ACC of 0.775 and test ACC of 0.774. Gradient boosting methods consistently outperformed traditional approaches, with RF and SVM showing moderate effectiveness with CV-ACC of 0.752 and 0.746, respectively. KNN demonstrated the poorest performance across all metrics with a CV-ACC of 0.618 and test ACC of 0.630, likely due to the high-dimensional nature of BLOSUM62 features and the complex non-linear relationships inherent in protein sequence data.

As shown in Table S7, the mouse dataset results ensured BiGKbhb strong performance, with the model achieving high performance metrics (CV-ACC of 0.835, RC of 0.868, PR of 0.815, MCC of 0.672, and AUC of 0.918) and robust test set generalization (ACC of 0.832, RC of 0.902, PR of 0.786, MCC of 0.672, and AUC of 0.902). Notably, the test set sensitivity reached 0.902, indicating excellent capability for identifying true positive Kbhb sites in mouse proteins.

CatBoost maintained its position as the best traditional classifier with cross-validation having ACC of 0.820, RC of 0.856, PR of 0.800, MCC of 0.642, and AUC of 0.906, though it experienced more significant performance degradation on the test set with ACC of 0.760 and AUC of 0.836. LightGBM showed improved relative performance on mouse data with CV-ACC of 0.807 and test ACC of 0.784, while XGBoost achieved comparable results with CV-ACC of 0.803 and test ACC of 0.768.

The performance gap between BiGKbhb and traditional classifiers is more pronounced in the mouse dataset. This gap may be attributed to the limited dataset size, which can amplify performance differences and reduce the reliability of generalization of traditional classifiers.

ِِAs shown in Table S8, the fungal dataset exhibited the most pronounced performance differences, with BiGKbhb consistently strong results that are better than those of all traditional approaches. BiGKbhb demonstrated exceptional cross-validation performance (ACC of 0.869, SN of 0.875, PR of 0.864, MCC of 0.740, and AUC of 0.944) with virtually identical test set metrics (ACC of 0.871, SN of 0.877, PR of 0.877, MCC of 0.742, and AUC of 0.945), indicating perfect generalization and model stability.

Among traditional classifiers, XGBoost and LightGBM tied for second place with cross-validation accuracies of 0.806 and 0.817, respectively, both achieving identical test accuracies of 0.830. XGBoost showed particularly strong test set performance with an AUC of 0.905, while LightGBM achieved an AUC of 0.914. CatBoost, despite being the top traditional classifier on human and mouse datasets, showed relatively lower performance on fungal data with CV-ACC of 0.811 and test ACC of 0.823.

**Fig. 6:**
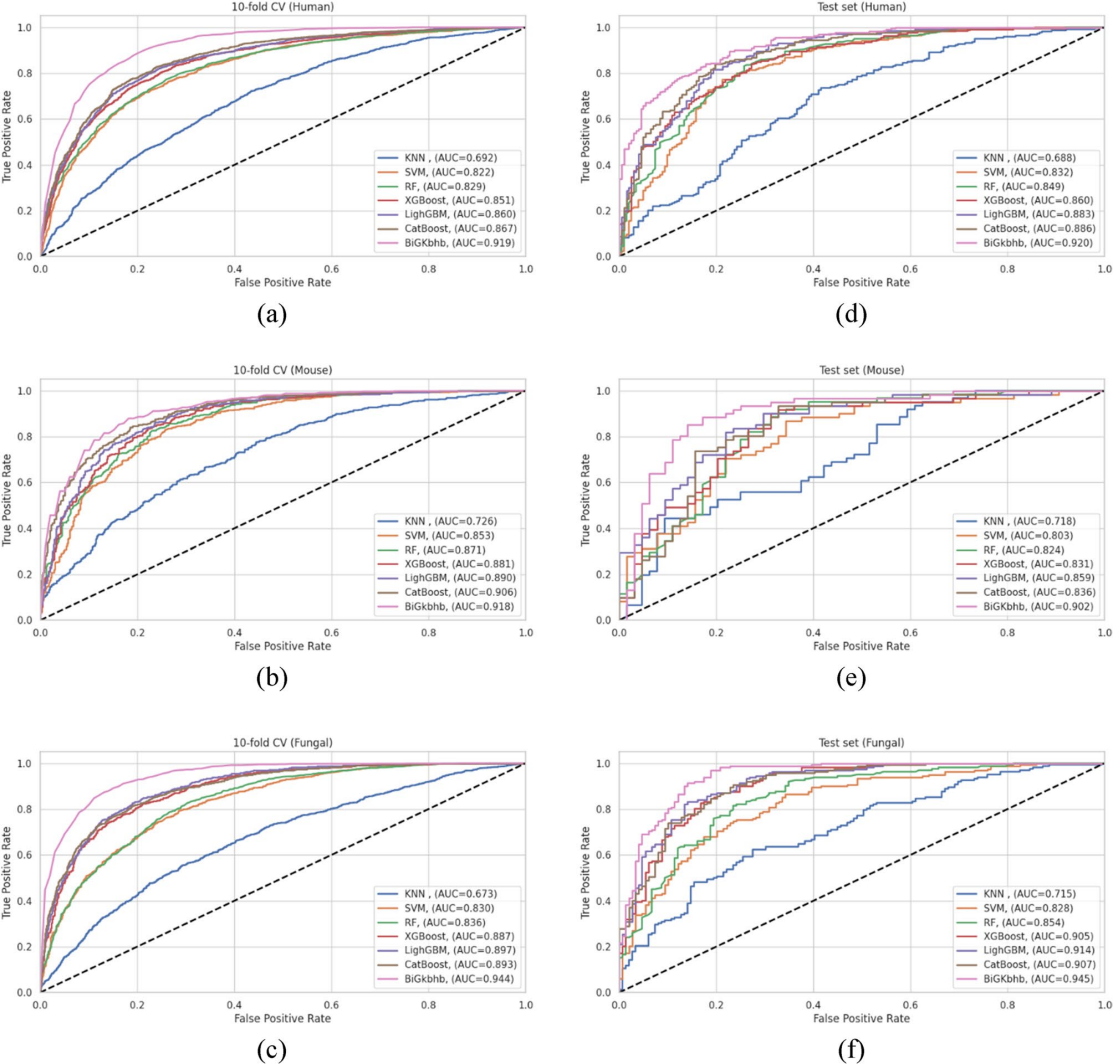
ROC curves comparing BiGKbhb with traditional machine learning classifiers across three species: (**a**-**c**) 10-fold cross-validation; and (**d**-**f**) independent test set performance for human, mouse, and fungal datasets, respectively

The ROC curves presented in Fig. 6 provide a comprehensive visualization of classifier performance across all three species, confirming the quantitative analysis. BiGKbhb consistently maintains the highest AUC values, demonstrating superior generalization capability. The curves clearly illustrate the performance hierarchy, with gradient boosting methods (CatBoost, LightGBM, and XGBoost) forming a distinct middle tier, traditional methods (RF, and SVM) showing moderate performance, and KNN consistently ranking lowest.

### Cross-species generalization assessment

Understanding how computational models perform across different species is crucial for advancing proteomics research, particularly when studying organisms with limited experimental data. In many cases, researchers need to apply prediction tools developed for well-studied species like humans to less-characterized organisms. To address this practical need, we evaluated whether our BiGKbhb model could effectively predict Kbhb sites across evolutionary boundaries and whether a single universal model might outperform species-specific approaches.

We designed a comprehensive evaluation comparing four distinct modeling strategies. To ensure proper training/testing separation and prevent data leakage, each species dataset was first split into training (90%) and independent test (10%) sets. The general model was then trained exclusively on the concatenated training portions from all three species, while species-specific models were trained only on their respective training sets. Importantly, evaluation was performed solely on the independent test sets that were never seen during training, ensuring unbiased assessment of model generalization capabilities. This experimental design allowed us to systematically assess both within-species performance and cross-species transferability across all model-dataset combinations.

The general model demonstrated remarkable robustness across all species tested, achieving consistently high performance with accuracy values ranging from 80% to 84% and AUC scores between 0.894 and 0.930 across human, mouse, and fungal datasets as shown in Table 9. This consistent performance ensures that fundamental sequence patterns governing Kbhb modification are conserved across evolutionary distances, enabling the development of broadly-applicable prediction tools. The balanced performance profile of the general model makes it particularly valuable for researchers working with diverse organisms or conducting comparative studies.

**Table 9:** Cross-species performance evaluation of the general BiGKbhb model trained on a concatenated multi-species dataset and tested on individual species-specific test sets

Test data	ACC	RC	PR	F1	MCC	AUC
General	0.844	0.855	0.840	0.847	0.687	0.918
Human	0.822	0.831	0.827	0.829	0.643	0.894
Mouse	0.800	0.738	0.833	0.783	0.603	0.920
Fungal	0.817	0.735	0.895	0.807	0.647	0.930

To further understand the contribution of individual species to the general model robust performance, we conducted a comprehensive ablation study evaluating all possible two-species combinations alongside the complete three-species model (Appendix B). This analysis revealed critical insights into optimal training data composition for cross-species Kbhb prediction.

**Fig. 7:**
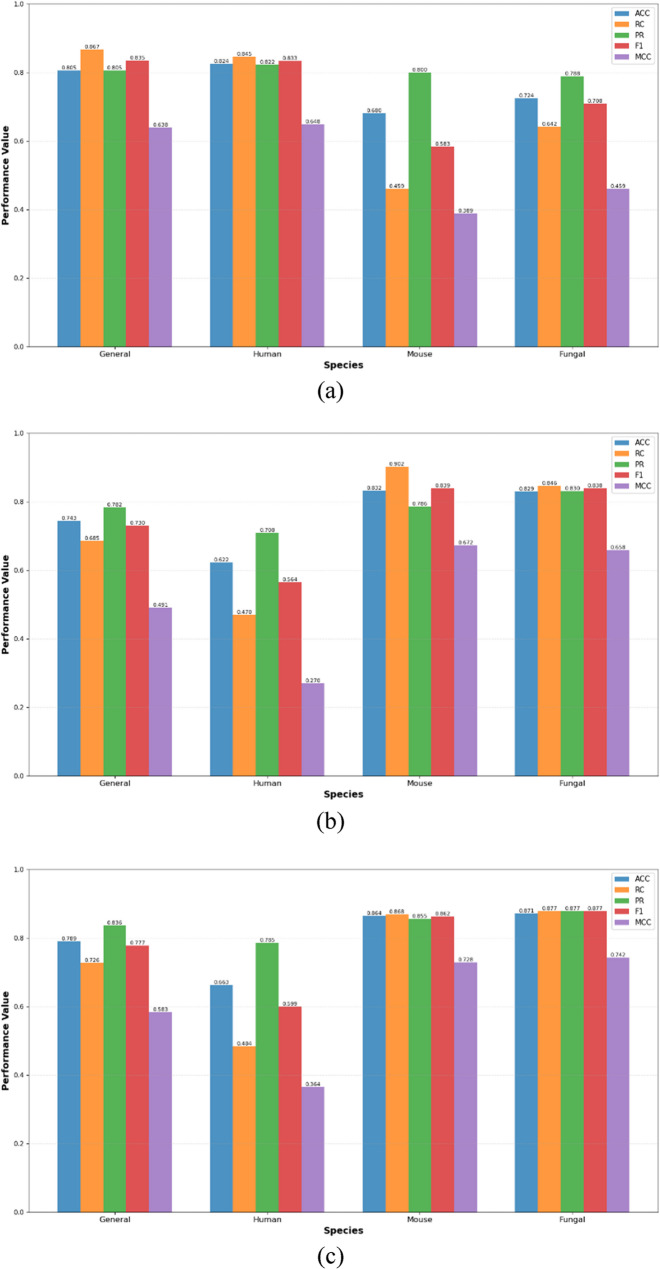
Cross-species transferability assessment of the (**a**) human, (**b**) mouse and (**c**) fungal-specific BiGKbhb model evaluated on general and species-specific test sets

Species-specific models revealed intriguing patterns of evolutionary conservation and divergence in Kbhb recognition mechanisms, as shown in Fig. 7. As shown in Fig. 7a, the human-specific model achieves an optimal performance on human proteins but shows a limited transferability to other species, particularly struggling with mouse proteins, where accuracy drops to 68%. This pattern confirms that human Kbhb sites possess unique sequence characteristics that may reflect species-specific adaptations in PTM machinery or regulatory networks.

The most surprising is the strong reciprocal transferability observed between mouse and fungal models. The mouse-specific model, as shown in Fig. 7b, maintains high performance when applied to fungal proteins with ACC of 83%; while the fungal model, as shown in Fig. 7c, achieves comparable success on mouse data with ACC of 86%. This unexpected compatibility between evolutionarily distant organisms reveals either convergent evolution in Kbhb modification mechanisms or the preservation of ancient regulatory patterns that predate the divergence of these lineages.

The transferability patterns provide important biological insights into the evolution of protein modification systems. The strong mouse-fungal compatibility, combined with the distinct human patterns, ensures that Kbhb recognition mechanisms may have undergone specific evolutionary changes in the human lineage. This finding aligns with our earlier motif analysis, which reveals that human proteins prefer small, flexible amino acids downstream of modification sites, while both mouse and fungal proteins favour larger hydrophobic residues in the same positions.

**Fig. 8:**
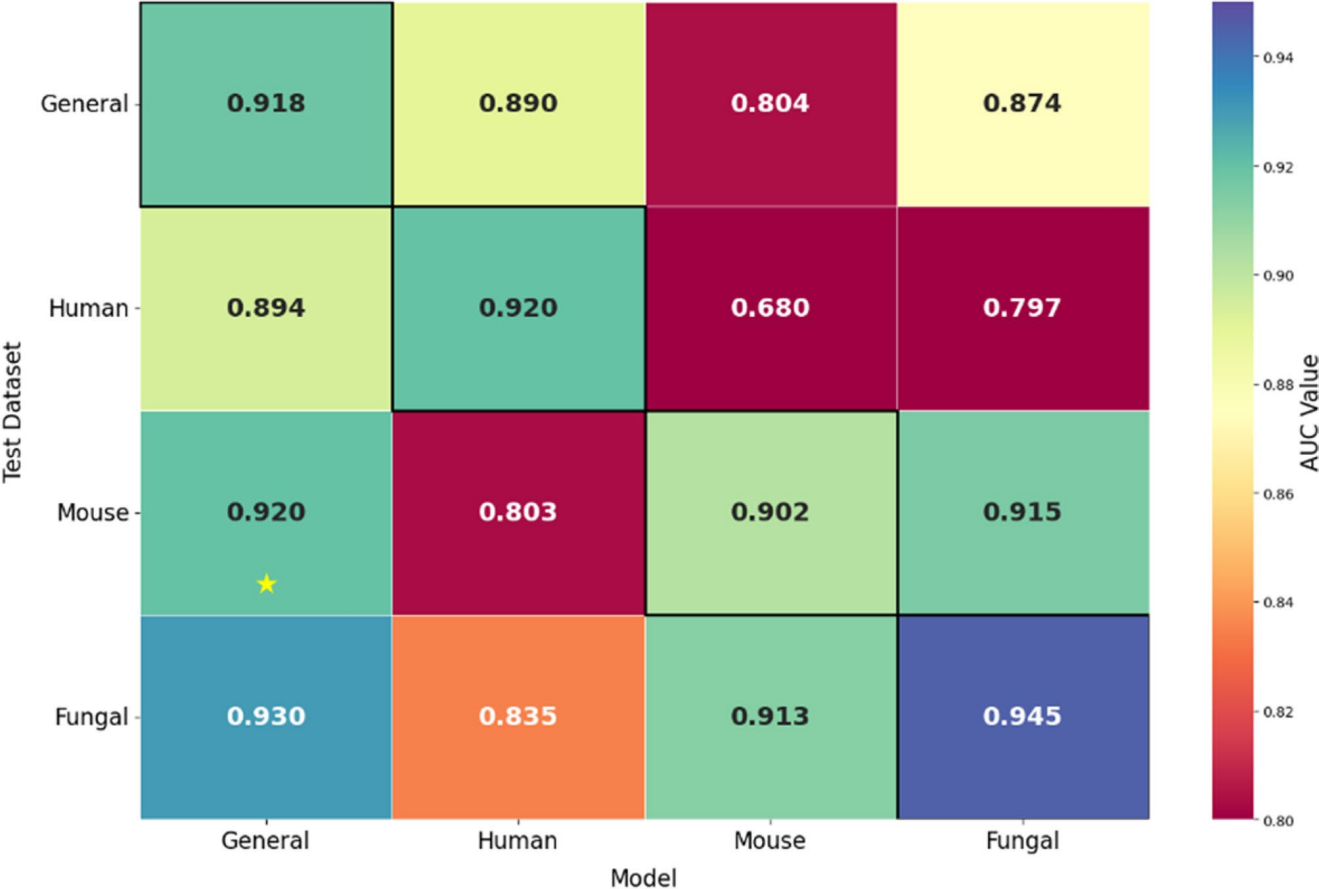
Heatmap visualization of AUC performance matrix showing cross-species generalization capabilities of general and species-specific BiGKbhb models across all test datasets. Yellow stars indicate cases where a non-native model outperforms the native model

The comprehensive AUC performance matrix shown in Fig. 8 reveals these cross-species transferability patterns, clearly demonstrating the consistent performance of the general model and the asymmetric transferability profiles of species-specific models.

From a practical perspective, these results strongly support the use of general models for Kbhb prediction across diverse species. The general model not only provides robust cross-species performance but occasionally outperforms species-specific models even on their native datasets, likely due to the enhanced training diversity that helps capture subtle but important sequence patterns. This finding has significant implications for proteomics research on understudied organisms, where species-specific training data may be limited or unavailable.

The cross-species analysis also validates our computational approach by demonstrating that the learned sequence patterns reflect real biological constraints rather than species-specific artifacts. The fact that models can successfully transfer knowledge across evolutionary boundaries indicates that our BiGRU architecture effectively captures the underlying biochemical principles governing Kbhb modification, providing confidence in predictions for novel or poorly characterized species.

#### Feature learning assessment through *t*-SNE visualization

**Fig. 9:**
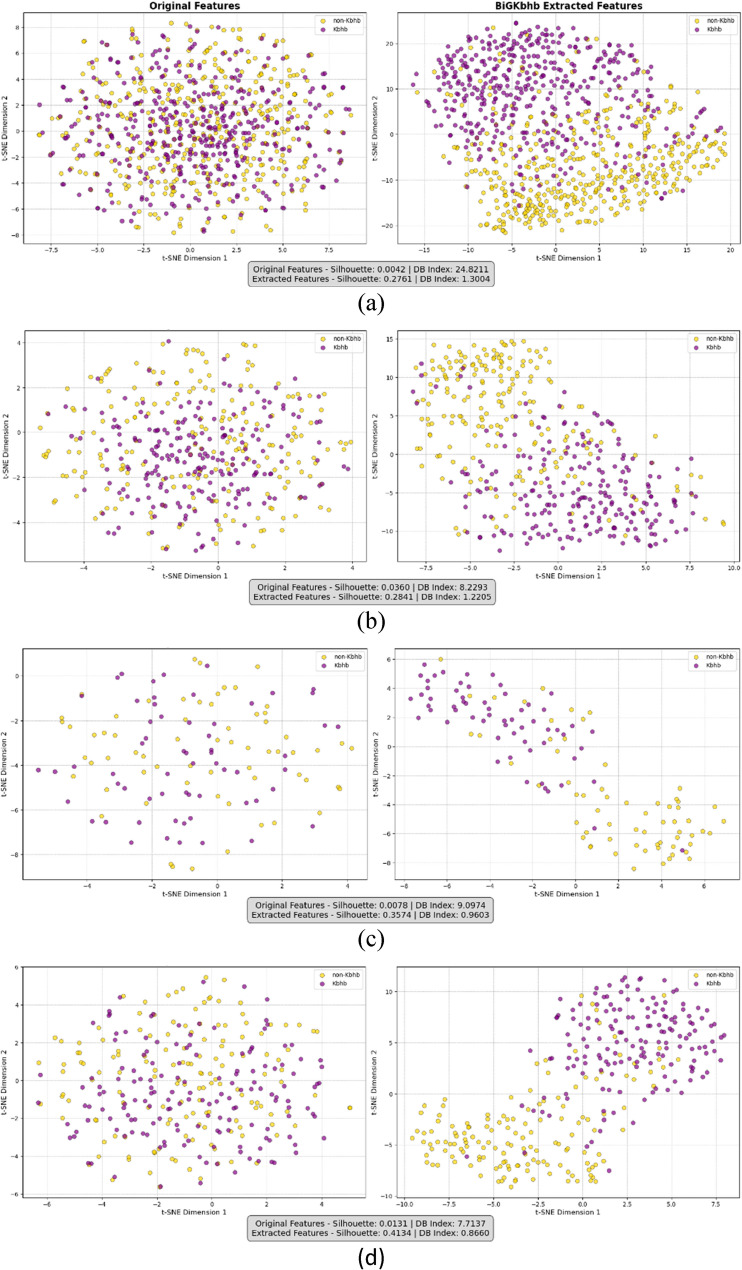
The *t*-SNE visualization of feature learning in BiGKbhb models: (**a**) general model, (**b**) human-specific model, (**c**) mouse-specific model, and (**d**) fungal-specific model. The left panels show input features, and the right panels show extracted features from the final hidden layer

We employed *t*-SNE to evaluate the feature learning capabilities of four BiGKbhb model variants by visualizing original BLOSUM62 input features and learned features extracted from the final hidden layer. Figure 9 presents paired visualizations for each model, where the left panel displays the original input features and the right panel shows the corresponding learned features. The analysis depends on standardized parameters, including perplexity of 30, a learning rate of 200, and 1000 iterations to ensure consistent comparisons.

To quantify clustering quality objectively, we calculated Silhouette Score [79], which measures intra-cluster cohesion and inter-cluster separation on a scale from − 1 to 1, where higher values indicate better-defined clusters. We used also the Davies-Bouldin (DB) Index [80] values, which reflect cluster validity by considering within-cluster scatter relative to between-cluster separation, where lower values indicate superior clustering performance.

The results demonstrated effective feature transformation capabilities across all model variants, with notable improvements from input to learned feature representations. The general BiGKbhb model transformed completely mixed input features with inferior clustering quality (Silhouette Score of 0.0042 and DB Index of 24.82) into learned features with clear vertical separation, achieving a 65.7-fold improvement in Silhouette Score to 0.2761 and reducing the DB Index to 1.30. The human-specific model demonstrated improvement from challenging input features (Silhouette Score of 0.0360 and DB Index of 8.23) to well-organized learned representations (Silhouette Score of 0.2841 and DB Index of 1.22), representing nearly an 8-fold enhancement in clustering quality despite the complexity of human sequence patterns.

The mouse-specific model exhibited strong feature transformation with input features showing extensive class mixing (Silhouette Score of 0.0078 and DB Index of 9.10) being converted into learned features with clear diagonal separation and the second-best clustering performance across all models (Silhouette Score of 0.3574 and DB Index of 0.96), representing a dramatic 45.8-fold improvement. The fungal-specific model achieved the most effective feature learning, transforming mixed input features (Silhouette Score of 0.0131 and DB Index of 7.71) into highly discriminative representations with pronounced horizontal separation and optimal clustering metrics (Silhouette Score of 0.4134 and DB Index of 0.87), demonstrating minimal class overlap and the highest clustering quality among all variants.

The comparative analysis revealed a clear performance hierarchy with the fungal-specific model achieving advanced clustering metrics, followed by mouse-specific, human-specific, and general models. The enhanced performance of species-specific models, particularly fungal and mouse variants, ensures that these organisms possess more distinctive and consistent Kbhb sequence patterns that the BiGRU architecture can effectively learn and exploit for accurate classification. The *t*-SNE analysis validates the BiGKbhb architecture robust feature transformation capabilities, with all models demonstrating improvements from input to learned features and confirming the architecture ability to extract meaningful discriminative patterns regardless of dataset complexity or species specificity, thereby validating both multi-species and species-specific training approaches for Kbhb site prediction.

#### Comparative performance evaluation against state-of-the-art Kbhb predictors

To assess the effectiveness of the proposed BiGKbhb model, we conducted comprehensive comparisons with the existing Kbhb site predictors: iBhb-Lys and KbhbXG. Both methods were reimplemented and retrained using our standardized datasets (human, mouse, fungal, and general) using both 10-fold cross-validation and independent test sets, to ensure fair and consistent evaluation across all approaches.

It should also be noted that SLAM, a recent structure-aware deep learning model for Kbhb prediction, was not included in our direct experimental comparison due to fundamental methodological differences. Specifically, SLAM requires 3D structural information to construct residue-level protein graphs and integrates both geometric and sequence features using a combination of GNNs, BiLSTMs, CNNs, and attention-based message passing. In contrast, BiGKbhb is designed for sequence-only prediction using fixed-length peptide windows. Although the two approaches address different computational contexts, we note that our model achieves comparable AUC values. Specifically, BiGKbhb attained test AUCs of 0.920 (human), 0.902 (mouse), and 0.945 (fungal), aligning closely with SLAM reported performance range (0.890–0.923), thereby demonstrating the strong predictive capability of our sequence-based framework.

The iBhb-Lys predictor represents a comprehensive approach to Kbhb site identification that combines multiple feature encoding strategies with dimensionality reduction and fuzzy classification techniques. As described in the original work, iBhb-Lys employs four distinct feature types to encode each Kbhb site as a 3266-dimensional feature vector, capturing various aspects of protein sequence characteristics. The method addresses the challenge of high-dimensional feature spaces through an autoencoder network for dimensionality reduction, followed by a novel fuzzy support vector machine algorithm that incorporates sample density into the fuzzy membership function to mitigate the effects of noise and outliers during classification. We reimplemented this approach using the same architectural principles and feature extraction methods as described in the original publication, training and evaluating the model on our standardized datasets to maintain consistency with our experimental framework.

The KbhbXG predictor depends on the XGBoost gradient boosting framework for Kbhb site prediction, representing a machine learning approach that leverages ensemble methods for classification. For our comparative analysis, we reimplemented the KbhbXG approach and optimized its hyperparameters using GridSearch cross-validation to ensure optimal performance on our datasets. The final XGBoost configuration employed a learning rate of 0.1, a maximum tree depth of 15, 500 estimators, and regularization parameters with *L*_1_ (α = 1) and *L*_2_ (λ = 2) penalties. This hyperparameter optimization process ensures that the KbhbXG implementation achieves its best possible performance for a fair comparison with our proposed BiGKbhb model.

The comparative evaluation results are presented in Table 10 for cross-validation performance and Table 11 for independent test set evaluation. The analysis reveals consistent performance patterns across all four datasets examined. On the human dataset, BiGKbhb achieved a cross-validation accuracy of 0.840 ± 0.013 compared to 0.775 ± 0.022 for KbhbXG and 0.767 ± 0.027 for iBhb-Lys. The test set performance showed similar trends, with BiGKbhb achieving 0.824 accuracy while KbhbXG and iBhb-Lys achieved 0.774 and 0.782, respectively. Notably, BiGKbhb demonstrated improved recall performance with an accuracy of 0.845 compared to both existing methods with accuracies of 0.795 for KbhbXG and 0.776 for iBhb-Lys, indicating enhanced sensitivity for identifying true positive Kbhb sites.

**Table 10:** Performance comparison of the BiGKbhb and existing Kbhb predictors on the 10-fold cross-validation sets. Boldface values indicate the best performance for each metric

Dataset	Predictor	ACC	RC	PR	MCC	AUC
Human	iBhb-Lys	0.767 ± 0.027	0.757 ± 0.038	0.772 ± 0.028	0.536 ± 0.053	0.837 ± 0.017
	KbhbXG	0.775 ± 0.022	0.764 ± 0.020	0.781 ± 0.028	0.551 ± 0.043	0.851 ± 0.019
	**BiGKbhb**	**0.840 ± 0.013**	**0.853 ± 0.028**	**0.830 ± 0.020**	**0.680 ± 0.026**	**0.920 ± 0.013**
Mouse	iBhb-Lys	0.736 ± 0.056	0.745 ± 0.084	0.732 ± 0.048	0.474 ± 0.113	0.810 ± 0.050
	KbhbXG	0.803 ± 0.043	0.854 ± 0.050	0.779 ± 0.054	0.612 ± 0.086	0.882 ± 0.027
	**BiGKbhb**	**0.835 ± 0.029**	**0.868 ± 0.039**	**0.815 ± 0.033**	**0.672 ± 0.059**	**0.919 ± 0.018**
Fungal	iBhb-Lys	0.714 ± 0.024	0.708 ± 0.041	0.715 ± 0.022	0.429 ± 0.047	0.791 ± 0.024
	KbhbXG	0.806 ± 0.027	0.823 ± 0.042	0.796 ± 0.033	0.614 ± 0.054	0.889 ± 0.030
	**BiGKbhb**	**0.869 ± 0.021**	**0.875 ± 0.045**	**0.864 ± 0.015**	**0.740 ± 0.043**	**0.945 ± 0.015**
General	iBhb-Lys	0.746 ± 0.016	0.741 ± 0.029	0.748 ± 0.013	0.493 ± 0.031	0.821 ± 0.012
	KbhbXG	0.784 ± 0.019	0.778 ± 0.020	0.787 ± 0.022	0.568 ± 0.037	0.861 ± 0.015
	**BiGKbhb**	**0.841 ± 0.013**	**0.863 ± 0.033**	**0.828 ± 0.017**	**0.684 ± 0.026**	**0.918 ± 0.007**

**Table 11:** Performance comparison of the BiGKbhb and existing Kbhb predictors on the test sets. Boldface values indicate the best performance for each metric

Dataset	Predictor	ACC	RC	PR	F1	MCC
Human	iBhb-Lys	0.782	0.776	0.798	0.787	0.563
	KbhbXG	0.774	0.795	0.777	0.786	0.548
	**BiGKbhb**	**0.824**	**0.845**	**0.833**	**0.833**	**0.648**
Mouse	iBhb-Lys	0.760	0.754	0.754	0.754	0.519
	KbhbXG	0.768	0.853	0.722	0.780	0.546
	**BiGKbhb**	**0.832**	**0.902**	**0.786**	**0.839**	**0.672**
Fungal	iBhb-Lys	0.724	0.685	0.760	0.721	0.451
	KbhbXG	0.830	0.870	0.815	0.842	0.659
	**BiGKbhb**	**0.871**	**0.877**	**0.877**	**0.877**	**0.742**
General	iBhb-Lys	0.739	0.697	0.769	0.731	0.482
	KbhbXG	0.803	0.766	0.833	0.798	0.608
	**BiGKbhb**	**0.844**	**0.855**	**0.840**	**0.847**	**0.687**

The mouse dataset results followed a similar pattern, with BiGKbhb achieving a test set ACC of 0.832 compared to 0.768 for KbhbXG and 0.760 for iBhb-Lys. The most pronounced performance differences were observed with the fungal dataset, where BiGKbhb reached 0.871 test ACC while KbhbXG and iBhb-Lys achieved 0.830 and 0.724, respectively. The general dataset, combining sequences from all three species, showed BiGKbhb achieving 0.844 accuracy compared to 0.803 for KbhbXG and 0.739 for iBhb-Lys.

**Fig. 10:**
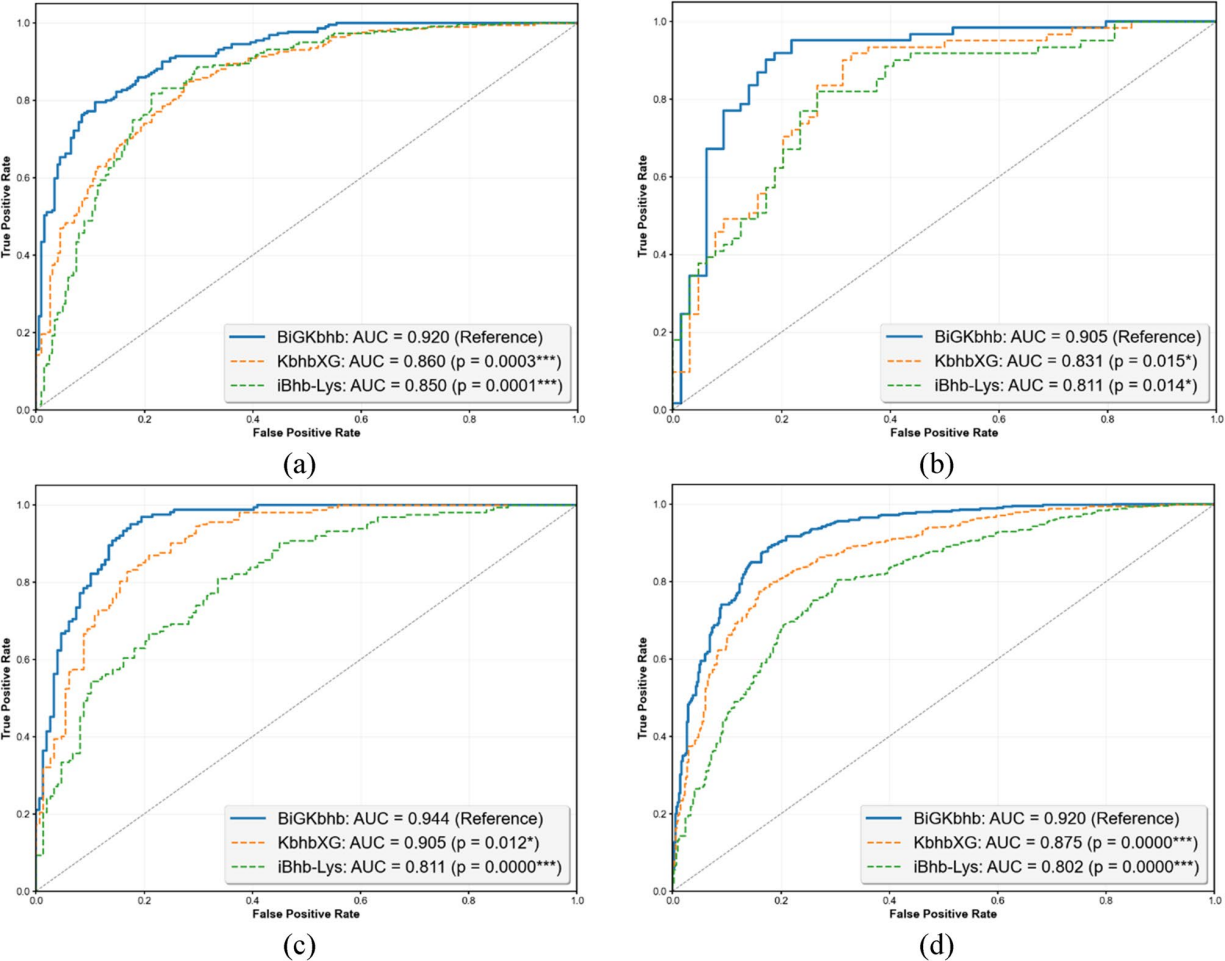
ROC curves demonstrating the comparative performance of BiGKbhb against existing Kbhb predictors across (**a**) Human, (**b**) Mouse, (**c**) Fungal, and (**d**) General datasets. Statistical significance levels are indicated as: *** *p* < 0.001, ** *p* < 0.01, * *p* < 0.05, ns = not significant (Bonferroni-corrected *p*-values from DeLong’s test)

The ROC curves presented in Fig. 10 provide visual confirmation of the performance differences across all datasets, demonstrating BiGKbhb consistently higher AUC values and improved classification capability across human, mouse, fungal, and general datasets.

Statistical analysis using DeLong’s test confirmed significant performance differences between BiGKbhb and existing methods. Across all datasets, the AUC differences between BiGKbhb and both existing methods reached statistical significance (*p* < 0.05). For the human dataset, BiGKbhb achieved an AUC of 0.9204 compared to 0.8599 for KbhbXG (difference of 0.0605, 95% Confidence Interval (CI) of [0.0291, 0.0919], *p* = 0.0003) and 0.8500 for iBhb-Lys (difference of 0.0704, 95% CI of [0.0366, 0.1042], *p* = 0.0001). The confidence interval represents the range of values within which we can be 95% confident that the true population difference in AUC values lies. Since no confidence interval includes zero, this provides additional evidence supporting the statistical significance of the observed performance improvements. Similar statistical significance was observed across mouse, fungal, and general datasets, with all comparisons showing corrected *p*-values well below the significance threshold.

The consistency between cross-validation and independent test set results provides insights into the generalization capabilities of each method. BiGKbhb demonstrates robust generalization with minimal performance degradation between cross-validation and test evaluation across all datasets. The MCC values, which provide balanced performance measures for binary classification, show BiGKbhb achieving values between 0.648 and 0.742 across datasets, compared to KbhbXG range of 0.546 to 0.659 and iBhb-Lys range of 0.451 to 0.563.

The computational efficiency analysis presented in Table 12 reveals important trade-offs between predictive performance and computational requirements. All measurements were conducted on the same hardware environment described above (AMD Ryzen 5 7520U, 2.80 GHz, 16.0 GB RAM) to ensure consistent and comparable timing assessments across all methods.

**Table 12:** Comparative analysis of computational efficiency metrics for Kbhb site prediction models across datasets. Boldface values indicate the best performance for each metric

Dataset	Method	Training time (sec)	Memory consumption (MB)	Inference speed/sample (sec)
Human	iBhb-Lys	30.44	**8.25**	0.709
	KbhbXG	**2.25**	45.73	**0.013**
	BiGKbhb	113.20	177.22	1.80
Mouse	iBhb-Lys	2.88	**6.51**	0.750
	KbhbXG	**0.875**	7.94	**0.012**
	BiGKbhb	49.25	48.22	1.07
Fungal	iBhb-Lys	20.45	40.58	0.458
	KbhbXG	**1.79**	**21.18**	**0.009**
	BiGKbhb	70.49	102.44	0.866
General	iBhb-Lys	156.18	87.10	3.15
	KbhbXG	**4.49**	**57.89**	**0.021**
	BiGKbhb	176.71	151.29	1.27

BiGKbhb requires substantially more computational resources during training, with training times ranging from 49.25 s (mouse dataset) to 176.71 s (general dataset), compared to KbhbXG 0.875 to 4.49 s across the same datasets. Memory consumption followed similar patterns, with BiGKbhb utilizing 48.22 to 177.22 MegaBytes (MB) compared to KbhbXG 7.94 to 57.89 MB. However, inference speed per sample showed BiGKbhb requiring 0.866 to 1.80 s compared to KbhbXG that is notably faster with 0.009 to 0.021 s.

The iBhb-Lys method demonstrated intermediate computational requirements, with training times between those of the two other approaches, but it is notably slower with inference speeds ranging from 0.458 to 3.15 s per sample. These computational considerations highlight the trade-off between the enhanced predictive accuracy achieved by BiGKbhb and the computational efficiency offered by traditional machine learning approaches like KbhbXG.

The computational efficiency trade-offs have important implications for different application scenarios. For high-throughput proteome-wide screening, where thousands of proteins need to be analyzed simultaneously, the faster inference of KbhbXG (0.009–0.021 s per sample) may be preferable despite lower accuracy, particularly when processing large datasets from mass spectrometry experiments. Conversely, BiGKbhb higher accuracy (6.5% improvement in the human dataset) justifies the increased computational cost (1.8 s per sample) in focused studies examining specific proteins or smaller datasets where precision is critical, such as drug target validation or biomarker discovery. The memory requirements also influence deployment scenarios: KbhbXG lower memory footprint (7.94–57.89 MB) makes it suitable for integration into automated pipelines or web server implementations with limited computational resources. On the other hand, BiGKbhb higher memory usage (48.22–177.22 MB) may require dedicated computational infrastructure, but it provides more reliable predictions for research applications, where false negatives could lead to missed biological insights. For clinical applications or real-time analysis scenarios, the choice between methods should consider whether the 6.5–8.7% accuracy improvement of BiGKbhb outweighs the 85–180-fold increase in inference time, depending on the specific requirements for sensitivity and throughput in the given application context.

## Conclusion

This study presented BiGKbhb, a novel deep learning framework that significantly advances computational prediction of Kbhb sites through systematic optimization of sequence encoding strategies and neural network architectures. Our comprehensive methodology identified BLOSUM62 evolutionary encoding combined with BiGRU architecture as optimal, demonstrating consistent improvements across all evaluated datasets and establishing new benchmarks for Kbhb site prediction. The research addresses a critical gap in PTM prediction tools by providing the first systematic evaluation of modern protein language models alongside traditional encoding approaches, revealing that evolutionary representations outperform sophisticated transformer-based embeddings for this specific modification type. Cross-species analysis demonstrated robust generalization capabilities, with the general model achieving enhanced transferability compared to species-specific approaches, while motif analysis unveiled distinct species-specific sequence preferences that provide insights into evolutionary adaptations of Kbhb modification mechanisms.

Although limitations include relatively-small dataset sizes, absence of experimental validation, context mismatch between pre-trained protein language models and our fixed peptide window approach, and computational efficiency trade-offs, this research establishes a robust foundation for future Kbhb prediction studies. The practical applications of this work extend to drug discovery and development, where accurate Kbhb prediction can identify novel therapeutic targets and biomarkers for metabolic diseases, cancer, and aging-related disorders. In precision medicine, these tools can facilitate personalized treatment strategies by predicting patient-specific modification patterns, while in pharmaceutical research, they can accelerate compound screening and reduce experimental costs by prioritizing the most promising candidates for wet lab validation. Additionally, the framework supports academic research by enabling proteome-wide identification of Kbhb sites in understudied organisms, facilitating comparative evolutionary studies, and advancing our understanding of metabolic regulation mechanisms. Future experimental validation through mass spectrometry, integration of structural information, comparison of full-sequence versus window-based PLM input strategies, expansion to additional species, and development of user-friendly web servers will enhance the translational impact and broader adoption of this computational approach in both research and clinical settings.

## Supplementary Information

Supplementary Material 1. Appendix A: Model Performance on Imbalanced Datasets.

Supplementary Material 2. Appendix B: Ablation Analysis of Species Contributions to Cross-Species Generalization**.**

Supplementary Material 3

## Data Availability

The protein accession numbers and the experimentally-validated Kbhb modification sites used in this study were compiled from the supplementary materials of three publications: human data from Huang et al. [54], mouse data from Hou et al. [55], and fungal (Ustilaginoidea virens) data from Chen et al. [56]. Corresponding peptide sequences were retrieved from the UniProt database (https://www.uniprot.org/) and the UniParc database (https://www.uniprot.org/uniparc) using the collected accession numbers. All data generated or analyzed during this study are included in this article.

## References

[1] Lee JM, et al. Control of protein stability by post-translational modifications. Nat Commun. 2023;14(1):201. 10.1038/s41467-023-35795-8. 36639369 10.1038/s41467-023-35795-8 PMC9839724

[2] Ye P, et al. Unlocking the brain’s code: the crucial role of post-translational modifications in neurodevelopment and neurological function. Phys Life Rev. 2025;53:187–214. 10.1016/j.plrev.2025.03.011. 40120399 10.1016/j.plrev.2025.03.011

[3] Peng Y, et al. Targeted protein posttranslational modifications by chemically induced proximity for cancer therapy. J Biol Chem. 2023. 10.1016/j.jbc.2023.104572. 36870680 10.1016/j.jbc.2023.104572 PMC10050664

[4] Ebert T, et al. Ageing – oxidative stress, PTMs and disease. Mol Aspects Med. 2022;86:101099. 10.1016/j.mam.2022.101099. 35689974 10.1016/j.mam.2022.101099

[5] Ramazi S, Zahiri J. Post-translational modifications in proteins: resources, tools and prediction methods. Database. 2021;2021:pbaab012. 10.1093/database/baab012. 10.1093/database/baab012 PMC8040245 33826699

[6] Leutert M, Entwisle SW, Villén J. Decoding post-translational modification crosstalk with proteomics. Mol Cell Proteomics. 2021. 10.1016/j.mcpro.2021.100129. 34339852 10.1016/j.mcpro.2021.100129 PMC8430371

[7] Stastna M. Post-translational modifications of proteins in cardiovascular diseases examined by proteomic approaches. FEBS J. 2025;292(1):28–46. 10.1111/febs.17108. 38440918 10.1111/febs.17108 PMC11705224

[8] Li X, et al. Post-translational modification of PTEN protein: quantity and activity. Oncol Rev. 2024. 10.3389/or.2024.1430237. 39144161 10.3389/or.2024.1430237 PMC11321960

[9] Sipilä KH, et al. Proline hydroxylation in collagen supports integrin binding by two distinct mechanisms. J Biol Chem. 2018;293(20):7645–58. 10.1074/jbc.RA118.002200. 29615493 10.1074/jbc.RA118.002200 PMC5961056

[10] Strowitzki MJ, Cummins EP. Taylor Protein hydroxylation by Hypoxia-Inducible factor (HIF) hydroxylases: unique or ubiquitous?. Cells. 2019;8. 10.3390/cells8050384. 10.3390/cells8050384 PMC6562979 31035491

[11] Azevedo C, Saiardi A. Why always lysine? The ongoing tale of one of the most modified amino acids. Adv Biol Regul. 2016;60:144–50. 10.1016/j.jbior.2015.09.008. 26482291 10.1016/j.jbior.2015.09.008

[12] Qin Z, et al. Current computational tools for protein lysine acylation site prediction. Brief Bioinform. 2024;25(6):bbae469. 10.1093/bib/bbae469. 39316944 10.1093/bib/bbae469 PMC11421846

[13] Zhang L, et al. DeepKhib: a deep-learning framework for lysine 2-hydroxyisobutyrylation sites prediction. Front Cell Dev Biol. 2020. 10.3389/fcell.2020.580217. 33015075 10.3389/fcell.2020.580217 PMC7509169

[14] Elreify HM, et al. An efficient machine-learning framework for predicting protein post-translational modification sites. Sci Rep. 2025;15(1):31179. 10.1038/s41598-025-13178-x. 40854916 10.1038/s41598-025-13178-x PMC12379237

[15] Larsen MR, et al. Analysis of posttranslational modifications of proteins by tandem mass spectrometry. Biotechniques. 2006;40(6):790–8. 10.2144/000112201. 16774123 10.2144/000112201

[16] Yakubu RR, Nieves E, Weiss LM. The methods employed in mass spectrometric analysis of posttranslational modifications (PTMs) and Protein-Protein interactions (PPIs). Adv Exp Med Biol. 2019;1140:169–98. 10.1007/978-3-030-15950-4_10. 31347048 10.1007/978-3-030-15950-4_10 PMC7059822

[17] English N, Torres M. Enhancing the discovery of functional Post-Translational ModificationPost-translational modification (PTM) sites with machine learning Models – Development, Validation, and interpretation. Computational methods for predicting Post-Translational modification sites. New York, NY: Springer US; 2022. pp. 221–60. 10.1007/978-1-0716-2317-6_12. D.B. Kc, Editor. 10.1007/978-1-0716-2317-6_12 35696084

[18] Hou T, et al. LAceP: lysine acetylation site prediction using logistic regression classifiers. PLoS ONE. 2014;9(2):e89575. 10.1371/journal.pone.0089575. 24586884 10.1371/journal.pone.0089575 PMC3930742

[19] Liu Y, Wang Q, Xi J. DeepDA-Ace: a novel domain adaptation method for species-specific acetylation site prediction. Mathematics. 2022. 10.3390/math10142364.

[20] Xie H, et al. Methyl-GP: accurate generic DNA methylation prediction based on a language model and representation learning. Nucleic Acids Res. 2025;53(6):gkaf223. 10.1093/nar/gkaf223. 40156859 10.1093/nar/gkaf223 PMC11952970

[21] Zeng W, Gautam A, Huson DH. MuLan-Methyl—multiple transformer-based language models for accurate DNA methylation prediction. Gigascience. 2023;12:giad054. 10.1093/gigascience/giad054. 10.1093/gigascience/giad054 PMC10367125 37489753

[22] Islam S, et al. MethEvo: an accurate evolutionary information-based methylation site predictor. Neural Comput Appl. 2024;36(1):201–12. 10.1007/s00521-022-07738-9.

[23] Huang C-H, et al. Ubisite: incorporating two-layered machine learning method with substrate motifs to predict ubiquitin-conjugation site on lysines. BMC Syst Biol. 2016;10(1):S6. 10.1186/s12918-015-0246-z. 10.1186/s12918-015-0246-z PMC4895383 26818456

[24] Fu H, et al. Deepubi: a deep learning framework for prediction of ubiquitination sites in proteins. BMC Bioinformatics. 2019;20(1):86. 10.1186/s12859-019-2677-9. 30777029 10.1186/s12859-019-2677-9 PMC6379983

[25] Malebary SJ, Rehman MSu, Khan YD. Icrotok-PseAAC: identify lysine crotonylation sites by blending position relative statistical features according to the chou’s 5-step rule. PLoS ONE. 2019;14(11):e0223993. 10.1371/journal.pone.0223993. 31751380 10.1371/journal.pone.0223993 PMC6874067

[26] Li Z, et al. Adapt-Kcr: a novel deep learning framework for accurate prediction of lysine crotonylation sites based on learning embedding features and attention architecture. Brief Bioinform. 2022;23(2):bbac037. 10.1093/bib/bbac037. 35189635 10.1093/bib/bbac037

[27] Qiao Y, Zhu X, Gong H. BERT-Kcr: prediction of lysine crotonylation sites by a transfer learning method with pre-trained BERT models. Bioinformatics. 2022;38(3):648–54. 10.1093/bioinformatics/btab712. 34643684 10.1093/bioinformatics/btab712

[28] Liu X, et al. Deep_Ksuccsite: a novel deep learning method for the identification of lysine succinylation sites. Front Genet. 2022. 10.3389/fgene.2022.1007618. 36246655 10.3389/fgene.2022.1007618 PMC9557156

[29] Ning W, et al. Hybridsucc: a hybrid-learning architecture for general and species-specific succinylation site prediction. Genomics Proteomics Bioinformatics. 2020;18(2):194–207. 10.1016/j.gpb.2019.11.010. 32861878 10.1016/j.gpb.2019.11.010 PMC7647696

[30] Pokharel S, et al. Improving protein succinylation sites prediction using embeddings from protein language model. Sci Rep. 2022;12(1):16933. 10.1038/s41598-022-21366-2. 36209286 10.1038/s41598-022-21366-2 PMC9547369

[31] Arafat ME, et al. Accurately predicting glutarylation sites using sequential bi-peptide-based evolutionary features. Genes. 2020. 10.3390/genes11091023. 32878321 10.3390/genes11091023 PMC7565944

[32] Ahmad MW et al. Improved performance of Lysine Glutarylation PTM using Peptide Evolutionary Features. in. 2019 3rd International Conference on Electrical, Computer & Telecommunication Engineering (ICECTE). 2019. 10.1109/ICECTE48615.2019.9303533.

[33] Dipta SR, et al. SEmal: accurate protein malonylation site predictor using structural and evolutionary information. Comput Biol Med. 2020;125:104022. 10.1016/j.compbiomed.2020.104022. 33022522 10.1016/j.compbiomed.2020.104022

[34] Ahmad W, et al. Mal-light: enhancing lysine malonylation sites prediction problem using evolutionary-based features. IEEE Access. 2020;8:77888–902. 10.1109/access.2020.2989713. 33354488 10.1109/access.2020.2989713 PMC7751949

[35] Xie Z, et al. Metabolic regulation of gene expression by histone lysine β-hydroxybutyrylation. Mol Cell. 2016;62(2):194–206. 10.1016/j.molcel.2016.03.036. 27105115 10.1016/j.molcel.2016.03.036 PMC5540445

[36] Ju Z, Zhang Q-B. iBhb-Lys: identify lysine β-hydroxybutyrylation sites using autoencoder feature representation and fuzzy SVM algorithm. Anal Biochem. 2025;697:115715. 10.1016/j.ab.2024.115715. 39521356 10.1016/j.ab.2024.115715

[37] Chen L, et al. KbhbXG: a machine learning architecture based on XGBoost for prediction of lysine β-Hydroxybutyrylation (Kbhb) modification sites. Methods. 2024;227:27–34. 10.1016/j.ymeth.2024.04.016. 38679187 10.1016/j.ymeth.2024.04.016

[38] Qin Z, et al. SLAM: structure-aware lysine β-hydroxybutyrylation prediction with protein language model. Int J Biol Macromol. 2024;280:135741. 10.1016/j.ijbiomac.2024.135741. 39293623 10.1016/j.ijbiomac.2024.135741

[39] Rives A, et al. Biological structure and function emerge from scaling unsupervised learning to 250 million protein sequences. Proc Natl Acad Sci U S A. 2021;118(15):pe2016239118. 10.1073/pnas.2016239118. 10.1073/pnas.2016239118 PMC8053943 33876751

[40] Gao Q, et al. Protein–protein interaction prediction model based on ProtBert-BiGRU-Attention. J Comput Biol. 2024;31(9):797–814. 10.1089/cmb.2023.0297. 39069885 10.1089/cmb.2023.0297

[41] Ferruz N, Schmidt S, Höcker B. ProtGPT2 is a deep unsupervised language model for protein design. Nat Commun. 2022;13(1):4348. 10.1038/s41467-022-32007-7. 35896542 10.1038/s41467-022-32007-7 PMC9329459

[42] Dubchak I, et al. Prediction of protein folding class using global description of amino acid sequence. Proc Natl Acad Sci U S A. 1995;92(19):8700–4. 10.1073/pnas.92.19.8700. 7568000 10.1073/pnas.92.19.8700 PMC41034

[43] Kawashima S, et al. AAindex: amino acid index database, progress report 2008. Nucleic Acids Res. 2008;36:D202–5. 10.1093/nar/gkm998. 17998252 10.1093/nar/gkm998 PMC2238890

[44] Eddy SR. Where did the BLOSUM62 alignment score matrix come from? Nat Biotechnol. 2004;22(8):1035–6. 10.1038/nbt0804-1035. 15286655 10.1038/nbt0804-1035

[45] Mohanasundaram R et al. Chap. 8 *-* Deep Learning and Semi-Supervised and Transfer Learning Algorithms for Medical Imaging, in Deep Learning and Parallel Computing Environment for Bioengineering Systems, A.K. Sangaiah, Editor. Academic Press. 2019; 139–151. 10.1016/B978-0-12-816718-2.00015-4.

[46] Nweke HF, et al. Deep learning algorithms for human activity recognition using mobile and wearable sensor networks: state of the art and research challenges. Expert Syst Appl. 2018;105:233–61. 10.1016/j.eswa.2018.03.056.

[47] Hochreiter S, Schmidhuber J. Long short-term memory. Neural Comput. 1997;9(8):1735–80. 10.1162/neco.1997.9.8.1735. 9377276 10.1162/neco.1997.9.8.1735

[48] Schuster M, Paliwal KK. Bidirectional recurrent neural networks. IEEE Trans Signal Process. 1997;45(11):2673–81.

[49] Cho K et al. Learning Phrase Representations using RNN Encoder-Decoder for Statistical Machine Translation. 2014. 10.48550/arXiv.1406.1078.

[50] Lynn H, Pan S, Kim P. A deep bidirectional GRU network model for biometric electrocardiogram classification based on recurrent neural networks. IEEE Access. 2019;1–1. 10.1109/ACCESS.2019.2939947.

[51] DeLong ER, DeLong DM, Clarke-Pearson DL. Comparing the areas under two or more correlated receiver operating characteristic curves: a nonparametric approach. Biometrics. 1988;44(3):837–45. 10.2307/2531595. 3203132

[52] Haynes W. Bonferroni correction. In: Dubitzky W, et al. editors. Encyclopedia of systems biology. New York: New York, NY: Springer; 2013. pp. 154–154. 10.1007/978-1-4419-9863-7_1213.

[53] Van der Maaten L, Hinton G. Viualizing data using t-SNE. J Mach Learn Res. 2008;9:2579–605. https://api.semanticscholar.org/. CorpusID:5855042

[54] Huang H, et al. The regulatory enzymes and protein substrates for the lysine β-hydroxybutyrylation pathway. Sci Adv. 2021. 10.1126/sciadv.abe2771. 33627428 10.1126/sciadv.abe2771 PMC7904266

[55] Hou W, et al. Quantitative proteomics analysis expands the roles of lysine β-hydroxybutyrylation pathway in response to environmental β-hydroxybutyrate. Oxid Med Cell Longev. 2022;2022(1):4592170. 10.1155/2022/4592170. 35251473 10.1155/2022/4592170 PMC8894020

[56] Chen X, et al. Post-Translational modification β-Hydroxybutyrylation Regulates < em > Ustilaginoidea virens Virulence. Mol Cell Proteom. 2023;22(8). 10.1016/j.mcpro.2023.100616. 10.1016/j.mcpro.2023.100616 PMC10423879 37442371

[57] Rossum Gv. Python Programming Language. in USENIX Annual Technical Conference. 2007. https://api.semanticscholar.org/. CorpusID:45594778.

[58] Consortium TU. UniProt: the universal protein knowledgebase in 2021. Nucleic Acids Res. 2020;49(D1):D480–9. 10.1093/nar/gkaa1100. 10.1093/nar/gkaa1100 PMC7778908 33237286

[59] Chen Z, et al. Large-scale comparative assessment of computational predictors for lysine post-translational modification sites. Brief Bioinform. 2019;20(6):2267–90. 10.1093/bib/bby089. 30285084 10.1093/bib/bby089 PMC6954452

[60] Fu L, et al. CD-HIT: accelerated for clustering the next-generation sequencing data. Bioinformatics. 2012;28(23):3150–2. 10.1093/bioinformatics/bts565. 23060610 10.1093/bioinformatics/bts565 PMC3516142

[61] Jia X, et al. ResNetKhib: a novel cell type-specific tool for predicting lysine 2-hydroxyisobutylation sites via transfer learning. Brief Bioinform. 2023. 10.1093/bib/bbad063. 36880172 10.1093/bib/bbad063 PMC10185920

[62] Pourmirzaei M, et al. Machine learning-based approaches for ubiquitination site prediction in human proteins. BMC Bioinformatics. 2023;24(1):449. 10.1186/s12859-023-05581-w. 38017391 10.1186/s12859-023-05581-w PMC10683244

[63] Arafat ME, et al. Accurate prediction of lysine methylation sites using evolutionary and structural-based information. Cogn Comput. 2024;16(3):1300–20. 10.1007/s12559-024-10268-2.

[64] Lin Z, et al. Evolutionary-scale prediction of atomic-level protein structure with a language model. Science. 2023;379(6637):1123–30. 10.1126/science.ade2574. 36927031 10.1126/science.ade2574

[65] Zeng W, et al. Improving prediction performance of general protein language model by domain-adaptive pretraining on DNA-binding protein. Nat Commun. 2024;15(1):7838. 10.1038/s41467-024-52293-7. 39244557 10.1038/s41467-024-52293-7 PMC11380688

[66] Suzek BE, et al. Uniref clusters: a comprehensive and scalable alternative for improving sequence similarity searches. Bioinformatics. 2015;31(6):926–32. 10.1093/bioinformatics/btu739. 25398609 10.1093/bioinformatics/btu739 PMC4375400

[67] Sarumi OA, Heider D. Large language models and their applications in bioinformatics. Comput Struct Biotechnol J. 2024;23:3498–505. 10.1016/j.csbj.2024.09.031. 39435343 10.1016/j.csbj.2024.09.031 PMC11493188

[68] Hallee L, Gleghorn JP. Protein-Protein Interaction Prediction is Achievable with Large Language Models. bioRxiv, 2023. 2023;06.07.544109. 10.1101/2023.06.07.544109.

[69] Kang H, et al. Fine-tuning of BERT model to accurately predict drug-target interactions. Pharmaceutics. 2022. 10.3390/pharmaceutics14081710. 36015336 10.3390/pharmaceutics14081710 PMC9414546

[70] Cabaneros SM, Hughes B. Methods used for handling and quantifying model uncertainty of artificial neural network models for air pollution forecasting. Environ Model Softw. 2022;158:105529. 10.1016/j.envsoft.2022.105529.

[71] Vacic V, Iakoucheva LM, Radivojac P. Two sample logo: a graphical representation of the differences between two sets of sequence alignments. Bioinformatics. 2006;22(12):1536–7. 10.1093/bioinformatics/btl151. 16632492 10.1093/bioinformatics/btl151

[72] O’Malley T et al. Keras tuner. 2019.

[73] Taunk K, et al. A Brief Review of Nearest Neighbor Algorithm for Learning and Classification. In: 2019 International Conference on Intelligent Computing and Control Systems (ICCS). 2019. 10.1109/ICCS45141.2019.9065747.

[74] Cortes C, Vapnik V. Support-vector networks. Mach Learn. 1995;20(3):273–97. 10.1007/BF00994018.

[75] Breiman L. Random forests. Mach Learn. 2001;45(1):5–32. 10.1023/A:1010933404324.

[76] Chen T, Guestrin C. XGBoost: A Scalable Tree Boosting System. 2016. 785–794. 10.1145/2939672.2939785.

[77] Ke G et al. LightGBM: a highly efficient gradient boosting decision tree. in Proceedings of the 31st International Conference on Neural Information Processing Systems. 2017. Long Beach, California, USA: Curran Associates Inc. https://www.api.semanticscholar.org/CorpusID:3815895.

[78] Prokhorenkova L et al. CatBoost: unbiased boosting with categorical features. in Proceedings of the 32nd International Conference on Neural Information Processing Systems. 2018. Montréal, Canada: Curran Associates Inc. 10.48550/arXiv.1706.09516.

[79] Shahapure KR, Nicholas C. 2020 IEEE 7th International Conference on Data Science and Advanced Analytics (DSAA). 2020. 10.1109/DSAA49011.2020.00096.

[80] Singh A et al. Clustering Evaluation by Davies-Bouldin Index(DBI) in Cereal data using K-Means. 2020. 306–310. 10.1109/ICCMC48092.2020.ICCMC-00057.

