# Supplementary material for: BiGKbhb: a bi-directional gated recurrent unit model for predicting lysine β-hydroxybutyrylation sites
Source: BMC Genomics. 2026 Jan 21;27:102. doi: 10.1186/s12864-025-12166-9 (PMC12836908; doi:10.1186/s12864-025-12166-9)
Supplement: Supplementary file 1 — Supplementary Material 1. Appendix A: Model Performance on Imbalanced Datasets. [file 12864_2025_12166_MOESM1_ESM.docx]

**Appendix A: Model Performance on Imbalanced Datasets**

To evaluate the robustness and real-world applicability of BiGKbhb under naturally occurring class imbalance conditions, we conducted additional experiments using the original imbalanced datasets without artificial balancing. These experiments address the practical scenario where negative samples (non-modified lysine sites) vastly outnumber positive samples (Kbhb-modified sites) in proteome-wide applications.

The imbalanced datasets maintained the natural distribution of positive and negative samples after clustering-based redundancy removal, as presented in Table A1. The class imbalance ratios varied significantly across species: the human dataset exhibited an 8.03:1 negative-to-positive ratio (16,900:2105), the mouse dataset showed a 6.10:1 ratio (3796:622), the fungal dataset demonstrated a 4.11:1 ratio (6372:1552), and the general dataset displayed the most severe imbalance with a 6.32:1 ratio (27,068:4279). These ratios reflect the realistic distribution encountered in computational proteomics applications, where modification sites represent a small fraction of all potential lysine residues.

| **Table A1.** Distribution of positive and negative samples in the imbalanced datasets. | | |
| --- | --- | --- |
| Dataset | Number of positive samples after clustering | Number of negative samples after clustering |
| Human | 2105 | 16,900 |
| Mouse | 622 | 3796 |
| Fungal | 1552 | 6372 |
| General | 4279 | 27,068 |

The BiGKbhb model was trained and evaluated using the same experimental protocol as the balanced datasets, employing 10-fold cross-validation and independent test set evaluation. Tables A2 and A3 present the comprehensive performance metrics for cross-validation and test set evaluations, respectively.

| **Table A2.** Performance of BiGKbhb on the 10-fold cross-validation of the imbalanced datasets | | | | | | |
| --- | --- | --- | --- | --- | --- | --- |
| **Dataset** | **ACC** | **RC** | **PR** | **F1** | **MCC** | **AUC** |
| Human | 0.922 ± 0.008 | 0.488 ± 0.086 | 0.719 ± 0.042 | 0.577 ± 0.06 | 0.551 ± 0.062 | 0.930 ± 0.009 |
| Mouse | 0.918 ± 0.012 | 0.648 ± 0.069 | 0.740 ± 0.046 | 0.690 ± 0.050 | 0.646 ± 0.054 | 0.943 ± 0.017 |
| Fungal | 0.902 ± 0.011 | 0.704 ± 0.055 | 0.776 ± 0.034 | 0.737 ± 0.034 | 0.679 ± 0.038 | 0.950 ± 0.010 |
| General | 0.901 ± 0.004 | 0.462 ± 0.042 | 0.715 ± 0.027 | 0.560 ± 0.028 | 0.523 ± 0.024 | 0.920 ± 0.007 |

| **Table A3.** Performance of BiGKbhb on the test sets of the imbalanced datasets. | | | | | | |
| --- | --- | --- | --- | --- | --- | --- |
| **Dataset** | **ACC** | **RC** | **PR** | **F1** | **MCC** | **AUC** |
| Human | 0.921 | 0.393 | 0.719 | 0.524 | 0.519 | 0.924 |
| Mouse | 0.923 | 0.774 | 0.706 | 0.739 | 0.695 | 0.959 |
| Fungal | 0.898 | 0.619 | 0.814 | 0.703 | 0.652 | 0.949 |
| General | 0.907 | 0.530 | 0.714 | 0.609 | 0.565 | 0.925 |

The evaluation of BiGKbhb on imbalanced datasets revealed distinct performance characteristics compared to the balanced dataset results presented in the main study. On the imbalanced datasets, the model achieved notably higher test accuracy values ranging from 0.898 to 0.923 across all species, compared to the balanced dataset test accuracies of 0.824 to 0.871. However, this apparent improvement in accuracy masks a significant trade-off in sensitivity, with recall values substantially lower on imbalanced data (0.393 to 0.774) compared to balanced data (0.845 to 0.902). This pattern reflects the well-documented phenomenon where models trained on imbalanced datasets tend to favour the majority class, achieving high overall accuracy by correctly predicting the abundant negative samples while struggling to identify the minority positive class.

The precision-recall trade-off analysis provides deeper insights into the model's behaviour under class imbalance conditions. Precision values remained relatively stable and competitive (0.706 to 0.814) across all datasets, indicating that when BiGKbhb predicts a positive Kbhb site, it maintains reasonable reliability. The F1-scores, which balance precision and recall, showed moderate values (0.524 to 0.739) that were consistently lower than those achieved on balanced datasets (0.833 to 0.877), reflecting the inherent challenge of maintaining both sensitivity and specificity under severe class imbalance. Notably, the test AUC values demonstrated remarkable stability (0.924 to 0.959), with some datasets even showing slight improvements compared to balanced conditions, confirming that the model's discriminative capability remains robust regardless of class distribution.

These findings have important implications for real-world applications of BiGKbhb in proteomics research. The high precision but lower recall observed on imbalanced datasets suggests that the model would be particularly valuable in scenarios where minimizing false positives is critical, such as expensive experimental validation studies where researchers need high confidence in predicted sites. However, for comprehensive proteome-wide screening applications where missing potential modification sites (false negatives) could overlook important biological insights, the balanced dataset approach may be more appropriate. The consistent AUC performance across both balanced and imbalanced conditions demonstrates the model's inherent quality and suggests that threshold optimization strategies could be employed to adjust the precision-recall trade-off based on specific application requirements.
