# Supplementary material for: BiGKbhb: a bi-directional gated recurrent unit model for predicting lysine β-hydroxybutyrylation sites
Source: BMC Genomics. 2026 Jan 21;27:102. doi: 10.1186/s12864-025-12166-9 (PMC12836908; doi:10.1186/s12864-025-12166-9)
Supplement: Supplementary file 2 — Supplementary Material 2. Appendix B: Ablation Analysis of Species Contributions to Cross-Species Generalization. [file 12864_2025_12166_MOESM2_ESM.docx]

**Appendix B: Ablation Analysis of Species Contributions to Cross-Species Generalization**

The species contribution ablation study (Table **B1**) provides valuable insights into the optimal training data composition for robust Kbhb site prediction across diverse organisms. The comprehensive analysis reveals that incorporating all three species (human, mouse, and fungal) yields the highest average performance (AUC: 0.915), demonstrating the value of multi-species training for developing generalizable prediction models. Notably, the human + fungal combination achieved equivalent average performance (AUC: 0.915), suggesting these two evolutionarily distant species provide complementary sequence patterns that effectively capture the diversity of Kbhb modification mechanisms.

The most striking finding concerns the critical importance of human data for cross-species generalization. When human data was excluded (mouse + fungal training), performance on human test data dropped dramatically to 0.759 AUC, representing a 15.1% decrease compared to the full model. This substantial performance degradation indicates that human Kbhb sites possess unique sequence characteristics that cannot be adequately captured by mouse and fungal data alone. Conversely, both human + mouse and human + fungal combinations maintained strong performance across all test datasets, suggesting that human data provides essential sequence diversity for robust generalization.

The superior performance of evolutionary distant species combinations (human + fungal) compared to closer relatives (human + mouse) supports the hypothesis that incorporating diverse evolutionary perspectives enhances model robustness. The fungal dataset appears particularly valuable, as its inclusion consistently improved cross-species performance, likely due to the distinct sequence patterns observed in our motif analysis (Figure 2c). These findings have practical implications for future Kbhb prediction tool development, indicating that strategic inclusion of evolutionarily diverse training data is more important than simply maximizing dataset size, and highlighting the continued importance of human data as a foundation for cross-species PTM prediction models.

| **Table B1.** Species contribution ablation study showing AUC performance of BiGKbhb models trained on different species combinations and evaluated across all species test datasets. | | | | |
| --- | --- | --- | --- | --- |
| Training data | Test on Human dataset | Test on Mouse dataset | Test on Fungal dataset | Average |
| All 3 species | 0.894 | 0.920 | 0.930 | 0.915 |
| Human + Mouse | 0.909 | 0.911 | 0.903 | 0.908 |
| Human + Fungal | 0.910 | 0.910 | 0.925 | 0.915 |
| Mouse + Fungal | 0.759 | 0.924 | 0.939 | 0.874 |
