# Supplementary material for: BiGKbhb: a bi-directional gated recurrent unit model for predicting lysine β-hydroxybutyrylation sites
Source: BMC Genomics. 2026 Jan 21;27:102. doi: 10.1186/s12864-025-12166-9 (PMC12836908; doi:10.1186/s12864-025-12166-9)
Supplement: Supplementary file 3 — Supplementary Material 3 [file 12864_2025_12166_MOESM3_ESM.docx]

**BiGKbhb: A Bi-directional Gated Recurrent Unit Model for Predicting Lysine Beta-Hydroxybutyrylation Sites.**

| **Table S1.** Optimized architectural parameters of BiGRU models across various peptide window sizes for Kbhb site prediction. | | | | | | | | | |
| --- | --- | --- | --- | --- | --- | --- | --- | --- | --- |
| **Window**  **size**  **Model**  **architecture** | **51** | **47** | **45** | **43** | **41** | **39** | **37** | **35** | **15** |
| Number of BiGRU layers | 1 | 1 | 1 | 1 | 1 | 1 | 1 | 1 | 1 |
| Number of units/layers | 192 | 192 | 256 | 192 | 192 | 128 | 192 | 64 | 32 |
| Batch normalization | 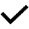 | 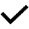 | 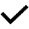 | 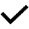 | 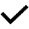 | 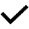 | 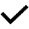 | 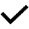 | 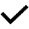 |
| Activation function | ReLU | ReLU | ReLU | ReLU | ReLU | ReLU | ReLU | ReLU | ReLU |
| Dropout/ layer | 0.4 | 0.4 | 0.3 | 0.4 | 0.5 | 0.3 | 0.3 | 0.1 | 0.1 |
| Global max pooling 1d | 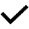 | 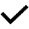 | 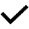 | 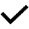 | 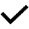 | 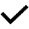 | 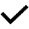 | 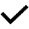 | 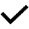 |
| Learning rate | 0.001 | 0.001 | 0.001 | 0.001 | 0.001 | 0.001 | 0.001 | 0.001 | 0.001 |

| **Table S2.** Optimized architectural parameters of deep learning models using BLOSUM62-encoded peptide sequences for Kbhb site prediction. | | | | | | |
| --- | --- | --- | --- | --- | --- | --- |
| **Model**  **Model**  **architecture** | **DNN** | **1DCNN** | **LSTM** | **BiLSTM** | **GRU** | **BiGRU** |
| Number of layers | 1 | 2 | 1 | 1 | 2 | 1 |
| Number of units/layers | 512 | 192  320 | 480 | 160 | 160  64 | 192 |
| Kernel size | - | 7  3 | - | - | - |  |
| Max pooling size/conv. layer | - | 3  4 | - | - | - |  |
| Batch normalization | 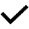 | 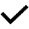 | 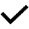 | 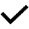 | 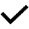 |  |
| Activation function | ReLU | ReLU | ReLU | ReLU | ReLU | ReLU |
| Number of dense layers | - | 5 | - | - | - | - |
| Number of units/dense layer | - | 192  352  224  224  128 | - | - | - | - |
| Dropout/ layer | 0.1 | 0.3  0.1  0.4  0.4  0.2 | 0.4 | 0.4 | 0.5  0.2 | 0.4 |
| Global max pooling 1d | 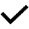 | - | 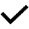 | 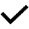 | 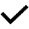 | 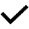 |
| Learning rate | 0.001 | 0.001 | 0.0001 | 0.001 | 0.001 | 0.001 |
| Number of epochs | 60 | 60 | 100 | 60 | 90 | 60 |

| **Table S3.** Comparative performance evaluation of deep learning architectures for Kbhb site prediction on the human dataset. | | | | | |
| --- | --- | --- | --- | --- | --- |
| **Model** | **10-fold cross-validation** | | | | |
|  | **ACC** | **RC** | **PR** | **MCC** | **AUC** |
| DNN | 0.724 ± 0.028 | 0.795 ± 0.073 | 0.698 ± 0.039 | 0.458 ± 0.054 | 0.817 ± 0.023 |
| 1DCNN | 0.769 ± 0.027 | 0.786 ± 0.044 | 0.761 ± 0.040 | 0.540 ± 0.053 | 0.844 ± 0.021 |
| GRU | 0.837 ± 0.019 | 0.827 ± 0.035 | 0.843 ± 0.024 | 0.675 ± 0.037 | 0.915 ± 0.011 |
| LSTM | 0.801 ± 0.026 | 0.823 ± 0.032 | 0.788 ± 0.031 | 0.604 ± 0.052 | 0.882 ± 0.022 |
| BiLSTM | 0.843 ± 0.015 | 0.848 ± 0.046 | 0.840 ± 0.031 | 0.688 ± 0.031 | 0.918 ± 0.012 |
| **BiGRU** | **0.840 ± 0.013** | **0.853 ± 0.028** | **0.830 ± 0.020** | **0.680 ± 0.026** | **0.919 ± 0.018** |
|  | **Test set** | | | | |
| DNN | 0.734 | 0.644 | 0.806 | 0.482 | 0.840 |
| 1DCNN | 0.772 | 0.739 | 0.806 | 0.545 | 0.852 |
| GRU | 0.831 | 0.872 | 0.816 | 0.663 | 0.907 |
| LSTM | 0.801 | 0.827 | 0.797 | 0.600 | 0.871 |
| BiLSTM | 0.812 | 0.822 | 0.818 | 0.624 | 0.894 |
| **BiGRU** | **0.824** | **0.845** | **0.833** | **0.648** | **0.920** |

| **Table S4.** Comparative performance evaluation of deep learning architectures for Kbhb site prediction on the mouse dataset. | | | | | |
| --- | --- | --- | --- | --- | --- |
| **Model** | **10-fold cross-validation** | | | | |
|  | **ACC** | **RC** | **PR** | **MCC** | **AUC** |
| DNN | 0.778 ± 0.037 | 0.822 ± 0.055 | 0.760 ± 0.050 | 0.560 ± 0.071 | 0.851 ± 0.042 |
| 1DCNN | 0.779 ± 0.037 | 0.824 ± 0.063 | 0.758 ± 0.036 | 0.563 ± 0.077 | 0.859 ± 0.037 |
| GRU | 0.813 ± 0.044 | 0.870 ± 0.063 | 0.791 ± 0.071 | 0.637 ± 0.080 | 0.888 ± 0.036 |
| LSTM | 0.725 ± 0.076 | 0.811 ± 0.122 | 0.701 ± 0.074 | 0.467 ± 0.140 | 0.816 ± 0.068 |
| BiLSTM | 0.839 ± 0.042 | 0.856 ± 0.039 | 0.832 ± 0.055 | 0.680 ± 0.083 | 0.919 ± 0.029 |
| **BiGRU** | **0.835 ± 0.029** | **0.868 ± 0.039** | **0.815 ± 0.033** | **0.672 ± 0.059** | **0.919 ± 0.018** |
|  | **Test set** | | | | |
| DNN | 0.744 | 0.754 | 0.730 | 0.488 | 0.810 |
| 1DCNN | 0.720 | 0.885 | 0.659 | 0.471 | 0.788 |
| GRU | 0.760 | 0.951 | 0.682 | 0.567 | 0.825 |
| LSTM | 0.776 | 0.902 | 0.714 | 0.573 | 0.874 |
| BiLSTM | 0.832 | 0.885 | 0.794 | 0.669 | 0.889 |
| **BiGRU** | **0.832** | **0.902** | **0.786** | **0.672** | **0.902** |

| **Table S5.** Comparative performance evaluation of deep learning architectures for Kbhb site prediction on the fungal dataset. | | | | | |
| --- | --- | --- | --- | --- | --- |
| **Model** | **10-fold cross-validation** | | | | |
|  | **ACC** | **RC** | **PR** | **MCC** | **AUC** |
| DNN | 0.747 ± 0.030 | 0.706 ± 0.075 | 0.768 ± 0.026 | 0.497 ± 0.055 | 0.844 ± 0.024 |
| 1DCNN | 0.730 ± 0.036 | 0.763 ± 0.045 | 0.714 ± 0.036 | 0.461 ± 0.071 | 0.800 ± 0.042 |
| GRU | 0.863 ± 0.024 | 0.877 ± 0.040 | 0.852 ± 0.025 | 0.727 ± 0.048 | 0.941 ± 0.010 |
| LSTM | 0.793 ± 0.081 | 0.824 ± 0.111 | 0.773 ± 0.072 | 0.590 ± 0.161 | 0.873 ± 0.090 |
| BiLSTM | 0.863 ± 0.017 | 0.871 ± 0.028 | 0.858 ± 0.030 | 0.727 ± 0.033 | 0.938 ± 0.013 |
| **BiGRU** | **0.869 ± 0.021** | **0.875 ± 0.045** | **0.864 ± 0.015** | **0.740 ± 0.043** | **0.945 ± 0.015** |
|  | **Test set** | | | | |
| DNN | 0.740 | 0.667 | 0.800 | 0.489 | 0.816 |
| 1DCNN | 0.724 | 0.747 | 0.729 | 0.446 | 0.783 |
| GRU | 0.871 | 0.907 | 0.855 | 0.743 | 0.933 |
| LSTM | 0.862 | 0.895 | 0.848 | 0.724 | 0.943 |
| BiLSTM | 0.868 | 0.901 | 0.854 | 0.737 | 0.936 |
| **BiGRU** | **0.871** | **0.877** | **0.877** | **0.742** | **0.945** |

| **Table S6.** Performance comparison between BiGKbhb and traditional machine learning classifiers for Kbhb site prediction in the human dataset. | | | | | |
| --- | --- | --- | --- | --- | --- |
| **Model** | **10-fold cross-validation** | | | | |
|  | **ACC** | **RC** | **PR** | **MCC** | **AUC** |
| KNN | 0.619 ± 0.019 | 0.860 ± 0.019 | 0.579 ± 0.015 | 0.274 ± 0.040 | 0.696 ± 0.022 |
| SVM | 0.746 ± 0.017 | 0.742 ± 0.027 | 0.746 ± 0.017 | 0.492 ± 0.034 | 0.822 ± 0.018 |
| RF | 0.740 ± 0.021 | 0.704 ± 0.043 | 0.756 ± 0.020 | 0.481 ± 0.041 | 0.809 ± 0.016 |
| XGBoost | 0.775 ± 0.022 | 0.764 ± 0.020 | 0.781 ± 0.028 | 0.551 ± 0.043 | 0.851 ± 0.019 |
| LightGBM | 0.785 ± 0.019 | 0.782 ± 0.024 | 0.786 ± 0.026 | 0.570 ± 0.038 | 0.860 ± 0.017 |
| CatBoost | 0.789 ± 0.016 | 0.776 ± 0.024 | 0.796 ± 0.026 | 0.579 ± 0.032 | 0.870 ± 0.015 |
| **BiGKbhb** | **0.840 ± 0.013** | **0.853 ± 0.028** | **0.830 ± 0.020** | **0.680 ± 0.026** | **0.919 ± 0.018** |
|  | **Test set** | | | | |
| KNN | 0.630 | 0.890 | 0.596 | 0.284 | 0.688 |
| SVM | 0.777 | 0.804 | 0.775 | 0.552 | 0.832 |
| RF | 0.777 | 0.808 | 0.773 | 0.553 | 0.849 |
| XGBoost | 0.774 | 0.795 | 0.777 | 0.548 | 0.860 |
| LightGBM | 0.808 | 0.836 | 0.803 | 0.615 | 0.883 |
| CatBoost | 0.822 | 0.840 | 0.821 | 0.643 | 0.886 |
| **BiGKbhb** | **0.824** | **0.845** | **0.833** | **0.648** | **0.920** |

| **Table S7.** Performance comparison between BiGKbhb and traditional machine learning classifiers for Kbhb site prediction in the mouse dataset. | | | | | |
| --- | --- | --- | --- | --- | --- |
| **Model** | **10-fold cross-validation** | | | | |
|  | **ACC** | **RC** | **PR** | **MCC** | **AUC** |
| KNN | 0.660 ± 0.053 | 0.791 ± 0.063 | 0.629 ± 0.048 | 0.331 ± 0.106 | 0.738 ± 0.055 |
| SVM | 0.782 ± 0.026 | 0.838 ± 0.056 | 0.756 ± 0.029 | 0.570 ± 0.056 | 0.855 ± 0.030 |
| RF | 0.791 ± 0.041 | 0.827 ± 0.053 | 0.775 ± 0.053 | 0.586 ± 0.083 | 0.863 ± 0.029 |
| XGBoost | 0.803 ± 0.043 | 0.854 ± 0.050 | 0.779 ± 0.054 | 0.612 ± 0.086 | 0.882 ± 0.027 |
| LightGBM | 0.807 ± 0.035 | 0.847 ± 0.045 | 0.787 ± 0.045 | 0.618 ± 0.069 | 0.891 ± 0.023 |
| CatBoost | 0.819 ± 0.032 | 0.856 ± 0.043 | 0.801 ± 0.049 | 0.643 ± 0.065 | 0.904 ± 0.023 |
| **BiGKbhb** | **0.835 ± 0.029** | **0.868 ± 0.039** | **0.815 ± 0.033** | **0.672 ± 0.059** | **0.919 ± 0.018** |
|  | **Test set** | | | | |
| KNN | 0.616 | 0.754 | 0.582 | 0.247 | 0.718 |
| SVM | 0.720 | 0.738 | 0.703 | 0.441 | 0.803 |
| RF | 0.768 | 0.820 | 0.735 | 0.540 | 0.824 |
| XGBoost | 0.768 | 0.853 | 0.722 | 0.546 | 0.831 |
| LightGBM | 0.784 | 0.836 | 0.750 | 0.573 | 0.859 |
| CatBoost | 0.760 | 0.820 | 0.725 | 0.526 | 0.836 |
| **BiGKbhb** | **0.832** | **0.902** | **0.786** | **0.672** | **0.902** |

| **Table S8.** Performance comparison between BiGKbhb and traditional machine learning classifiers for Kbhb site prediction in the fungal dataset. | | | | | |
| --- | --- | --- | --- | --- | --- |
| **Model** | **10-fold cross-validation** | | | | |
|  | **ACC** | **RC** | **PR** | **MCC** | **AUC** |
| KNN | 0.623 ± 0.022 | 0.720 ± 0.025 | 0.602 ± 0.022 | 0.251 ± 0.043 | 0.673 ± 0.019 |
| SVM | 0.748 ± 0.032 | 0.765 ± 0.045 | 0.739 ± 0.035 | 0.497 ± 0.064 | 0.831 ± 0.032 |
| RF | 0.747 ± 0.035 | 0.740 ± 0.053 | 0.751 ± 0.039 | 0.496 ± 0.069 | 0.825 ± 0.035 |
| XGBoost | 0.806 ± 0.027 | 0.823 ± 0.042 | 0.796 ± 0.033 | 0.614 ± 0.054 | 0.889 ± 0.030 |
| LightGBM | 0.817 ± 0.035 | 0.826 ± 0.047 | 0.812 ± 0.037 | 0.636 ± 0.069 | 0.898 ± 0.029 |
| CatBoost | 0.811 ± 0.034 | 0.809 ± 0.044 | 0.811 ± 0.043 | 0.622 ± 0.067 | 0.894 ± 0.027 |
| **BiGKbhb** | **0.869 ± 0.021** | **0.875 ± 0.045** | **0.864 ± 0.015** | **0.740 ± 0.043** | **0.945 ± 0.015** |
|  | **Test set** | | | | |
| KNN | 0.640 | 0.741 | 0.632 | 0.278 | 0.715 |
| SVM | 0.743 | 0.772 | 0.744 | 0.484 | 0.828 |
| RF | 0.781 | 0.759 | 0.809 | 0.564 | 0.854 |
| XGBoost | 0.830 | 0.870 | 0.815 | 0.659 | 0.905 |
| LightGBM | 0.830 | 0.870 | 0.815 | 0.659 | 0.914 |
| CatBoost | 0.823 | 0.827 | 0.832 | 0.646 | 0.907 |
| **BiGKbhb** | **0.871** | **0.877** | **0.877** | **0.742** | **0.945** |
